# The first precinctive Carabidae from Moorea, Society Islands: new *Mecyclothorax* spp. (Coleoptera) from the summit of Mont Tohiea

**DOI:** 10.3897/zookeys.224.3675

**Published:** 2012-09-29

**Authors:** James K. Liebherr

**Affiliations:** 1 Cornell University Insect Collection, Department of Entomology, John H. and Anna B. Comstock Hall, Cornell University, Ithaca, NY 14853-2601, USA

**Keywords:** French Polynesia, Moriomorphini, adaptive radiation, biogeography, colonization

## Abstract

Seven species of *Mecyclothorax* Sharp from Moorea, Society Islands are newly described; *Mecyclothorax perraulti* **sp. n.**, *Mecyclothorax pahere* **sp. n.**, *Mecyclothorax menemene* **sp. n.**, *Mecyclothorax mahatahi* **sp. n.**, *Mecyclothorax popotioaoa* **sp. n.**, *Mecyclothorax mapo* **sp. n.**, and *Mecyclothorax fatata* **sp. n.** These constitute the first *Mecyclothorax* species described from Moorea, and the first carabid beetle species shown to be geographically restricted to that island. Each of the newly described species is most similar to a different species on the island of Tahiti, suggesting that none of the seven Moorean taxa are evolutionary end-products of autochthonous speciation within Moorea. The occurrence of precinctive *Mecyclothorax* species on both Moorea and Tahiti demonstrates that radiation of *Mecyclothorax* in the Society Islands has been facilitated by speciation events implicating both islands. Whether this speciation has been preceded by vicariance or dispersal is discussed, with the generality of a dispersal hypothesis tested using information from Society Island Nabidae (Hemiptera). Salient morphological characters for taxa in the Society and Hawaiian Islands are compared to those representing a broad survey of southwest Pacific *Mecyclothorax* spp. This comparison supports the independent founding of each radiation in the Societies and Hawaii from an Australian ancestral propagule, likely drawn from the ecologically general, geographically widespread *Mecyclothorax punctipennis* (Macleay).

## Introduction

The genus *Mecyclothorax* Sharp is distributed throughout Australia and associated landmasses and islands including New Guinea, the Greater Sundas of Java and Borneo, Lord Howe and Norfolk Islands, and St. Paul and Amsterdam Islands of the Indian Ocean (Baehr 1998; Baehr and Lorenz 1999; Moore 1985, 1992). *Mecyclothorax* has also diversified in New Caledonia (Jeannel 1943; Deuve 1987) and New Zealand (Liebherr and Marris 2009). But it is on two Polynesian archipelagoes that *Mecyclothorax* has undergone radiations that are so rich in species that these radiations dwarf the levels of diversity observed over all other areas of the generic distribution. The Hawaiian Islands house a *Mecyclothorax* fauna that includes 166 valid species (Liebherr 2006, 2007, 2008, 2009a, 2009b, 2011a; Liebherr and Krushelnycky 2011), with an estimated 73 additional species in the process of description from Haleakala volcano, Maui Ialand (unpubl. data). *Mecyclothorax* beetles are distributed on the Hawaiian islands of Oahu, Molokai, Lanai, Maui and Hawaii Island, but not Kauai. The Society archipelago, specifically Tahiti, also supports an impressively diverse radiation, with 67 species recognized from the island of Tahiti (Perrault 1992). Why have these two Polynesian archipelagoes been home to such diverse *Mecyclothorax* radiations? Based on shared attributes of Tahiti and the Hawaiian Islands, *Mecyclothorax* have thrived in these places in association with subtropical montane rain forest that is dissected by lava flows or by low-elevation erosionally formed valleys, with the islands’ orographic relief resulting in extensively subdivided habitats ranging from 1000–3000 m elevation. In Tahiti Perrault (1992) discovered that different, neighboring mountain ridges emanating from the central peak Mont Orohena mostly supported distinct species. Though some species are shared between adjacent ridges—e.g. the ridges culminating at Mont Marau and Mont Aorai—the majority of species are not so shared between different ridges (Perrault 1992: 211). These montane habitats receive anywhere from 4000–6000 mm of precipitation per year in Hawaii (Giambelluca and Schroeder 1998) and up to 8000 mm/yr in Tahiti (Mueller–Dombois and Fosberg 1998; Craig et al. 2001). That *Mecyclothorax* speciation is facilitated by the geological subdivision of wet to mesic montane forest habitats is strongly supported by the geographic restriction of the vast majority of species in both radiations to rain forest habitats. The high levels of diversity in these subtropical islands contrasts sharply with the low *Mecyclothorax* diversity resident in Australia and New Zealand (Moore et al. 1987; Liebherr and Marris 2009), where species distributions are centered on open habitats including grasslands, moorlands, riparian corridors, and dry to mesic *Eucalyptus* forest.

**Figure 1.**
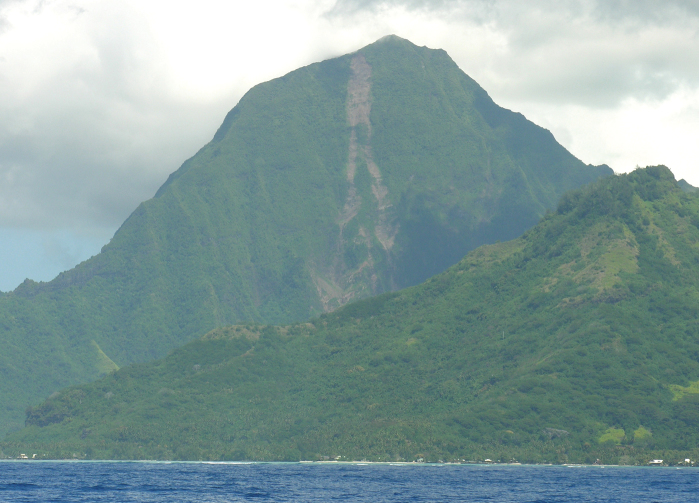
Mont Tohiea, Moorea, east face. Collections of *Mecyclothorax* spp. have been limited to the highest portions of the summit ridge, from 1100 m to the summit, elevation 1207 m.

This paper extends the comparison of the Hawaiian and Tahitian *Mecyclothorax* radiations by describing the first collections of *Mecyclothorax* species from a second Society Island, Moorea. Whereas Tahiti, including the volcanoes Tahiti Nui and Tahiti Iti, or Presqu’île de Taiarapu, encompasses 1040 km^2^, with highest elevations of 2241 m and 1332 m respectively on the two constituent volcanoes, Moorea has a much smaller land area of 142 km^2^ and a peak elevation of 1207 m at the summit of Mont Tohiea (Fig. 1). Rainfall is also less abundant on Moorea, though the 5000 mm/yr recorded precipitation (Craig et al. 2001) is similar to that observed in many windward areas of Hawaii that house distributions of Hawaiian *Mecyclothorax*. Hypotheses of sister-group relationships for each of the new Moorean species are proposed based on morphological characteristics, with independent relationships to different Tahitian species posited for each of the Moorean species. Therefore the faunas of these two islands that are currently separated by 23 km of ocean are closely related biogeographically, though there has been sufficient isolation—as predicted by the extreme endemism previously reported on Tahiti—to have resulted in *Mecyclothorax* faunas on the two islands that are absolutely distinct at the species level. Characters presented by taxa across the Society Island *Mecyclothorax* radiation distinctively differ from those characterizing the more generalized members of the Hawaiian *Mecyclothorax* radiation, supporting independent colonization events from Australia and subsequent adaptive radiations for each of these lineages on their particular archipelagoes.

## Methods

### Taxonomic material

Type specimens of Tahitian *Mecyclothorax* species used in the diagnosis of the new species were borrowed from the Naturhistorisches Museum, Basel (NHMB) and the Muséum national d’Histoire naturelle, Paris (MNHN). Primary type specimens of the new Moorean species, and associated allotypic paratypes where available, are deposited in the MNHN and incorporated into the Georges G. Perrault collection of Tahitian Carabidae. Other institutional depositories include: Cornell University Insect Collection (CUIC); Essig Museum of Entomology Collection (EMEC); U.S. National Museum of Natural History (NMNH).

### Laboratory techniques

Specimens were killed in ethyl acetate impregnated killing jars, maintained under that atmosphere for 24 hr, and then transferred to 70% ethanol for transport to the laboratory, or preserved directly in 100% ethanol. The former specimens were pointed or platen-mounted from ethanol. The latter specimens were maintained in ethanol at -16˚ C and examined under ethanol at room temperature, or removed from ethanol and pointed when a specimen was required for a holotype or allotype specimen. Description of characters was based only on air-dried specimens viewed using a dissecting microscope under bidirectional halogen light.

Labeling is presented verbatim for holotype and associated allotype specimens. Individual lines on labels are separated by a single slash ( / ), and separate labels are indicated by a double slash ( // ). Data for the other paratypes is presented in a standardized, condensed format organized chronologically by collection. Where information is repeated in adjacent, subsequent collections, that field is removed from the data string and is to be interpolated from the previous paratype data entry. Paratypes labeled with an MBIO#### lot number were retained in 100% ethanol and returned to EMEC for ensuing DNA extraction.

Dissection, clearing and staining techniques follow Liebherr (2009a). Genitalic dissections are maintained with the specimens in polyethylene genitalia vials. Photographs were made using a Microptics® (now Visionary Digital®) macrophotographic apparatus, including strobe flash illumination conveyed via fiber-optic wands, and a transmissible light stage. Habitus photos were taken using transmitted and reflected light, with specimens mounted on microscope slides and surrounded with two internested plexiglass rings lined with translucent Mylar® film. Genitalic dissections were photographed using transmitted light.

Descriptive conventions build upon data reported by Perrault (1978a, 1978b, 1984, 1986, 1987, 1988, 1989), with both standardized body length and setal formula presented in each diagnosis. The former consists of the sum of three measurements: 1, the distance from the anteriormost labral margin to the cervical ridge, estimated when necessary by extrapolation from the lateral reaches of the ridge; 2, the median length of the pronotum; 3, the distance from the base of the scutellum, where the scutellar dorsal surface dips ventrally, to the apex of the longer elytron, measured parallel to the suture. The setal formula (e.g., 1234) is based on the number of setae on one side of the beetle, with the four numbers signifiying: 1, the number of supraorbital setae, either one or two; 2, the number setae along the lateral margin of the pronotum, either one or two; 3, the number of dorsal elytral setae in the discal portion of the third elytral interval, ranging from 0–3 in Tahitian *Mecyclothorax*; and 4, the number of setae at the apex of the elytra. There are either one or two supraorbital setae; if one is absent it is the anterior seta. There are either one or two pronotal setae; if one is absent, the loss occurs at the setal position near the hind angle. There may be one or two dorsal elytral setae (or three unilaterally in one instance herein), and if a seta is lost evolutionarily, it is lost from the more apical position. Plesiomorphically there are two setae near the elytral apex, an apical seta near the apex of the second elytral stria along the elytral margin, and a subapical seta located in the seventh elytral stria anywhere from dorsad the subapical sinuation to closer to the apex of stria 7. Perrault (1978a et. seq.) reported the number of setae near the elytral apex without regard to their homology. In Moorean *Mecyclothorax*, the apical seta is always present; the subapical may be present or absent.

Various ratios are used to characterize shapes or relative dimensions. The ocular ratio is the measurement across the outer surface of the compound eyes divided by the minimum distance across the frons between the eyes. The ocular lobe ratio is the distance from the anterior to posterior margin of the eye measured from directly above, divided by the distance from the anterior margin of the eye to the groove at the juncture of the gena and ocular lobe using the same viewpoint. Various body meaurements are presented as ratios, with the measurements including: APW, anterior pronotal width, i.e. the width between the most anteriormost pronotal margins at the front angles; MPW, maximum pronotal width; BPW, basal pronotal width measure across the hind angles; PL, median pronotal length; HuW, humeral width, or distance between the anteriormost points along the basal groove-humeral juncture, i.e., the humeral angle; MEW, maximum elytral width. In order to present infraspecific variation in body shape, a maximum of five specimens, where available, were measured to compose these ratios. All available specimens were scanned and the largest individual, the smallest individual, and representatives of both sexes were included in the sample of five. Each of these specimens was labeled with a small number label that corresponds to its entry in the character matrix (unpubl. data). This sampling produced a range of ratios, with the smallest and largest individuals often producing the most disparate ratios. The number of sampled individuals, which may range from 1 to 5, is presented as (n = X).

The configuration of the antennae—moniliform, submoniliform, filiform, or elongate filiform—is categorized using a ratio of the dimensions of the eighth antennomere; length from basal juncture with seventh antennomere to apex divided by the maximal breadth, excluding the dense setal pelage. The configuration of the metathorax is quantified by the metepisternum width/length ratio. Width is measured perpendicular to the longitudinal body axis from the lateral edge adjacent to elytral epipleuron, to the medial juncture of mesepimeron, metasternum and metepisternum. Length is measured as the length of the medial edge from mesepimeron to juncture with metepimeron.

Elytral setation is described based on the dorsal setal positions in the third elytral interval relative to overall elytral length as measure in the standardized body length. The lateral elytral setae of the ninth interval, just laterad the eighth stria, are arrayed in an anterior series starting laterad the humerus, and an apical series that terminates just anterad the subapical sinuation. The two series are presented as A + B, with variation in setal number among individuals reported in parentheses; (B – C). When a particular setal count varied, and one state was observed only rarely, the rarely observed setal number is presented alone in parentheses; i.e., B(C).

Coloration is graded relatively from flavous (i.e. yellow without melanization), to rufoflavous, and then to rufobrunneous. Colors darker than rufobrunneous may entail dominant reddish coloration, thereby leading to rufous, dark rufous, and rufopiceous, or the colors may be dominated by browns, leading from rufobrunneous to brunneous, to rufopiceous. The darkest coloration observed is piceous, or shiny coal black. These base colors may be modified by a darker cast; incomplete melanization of the surface near setae or in thicker portions of the cuticle.

Description of microsculpture follows the general terms used in Lindroth (1974). A mature male specimen was assessed when available, though no consistent differences were observed between male and female specimens; far greater differences were observable when comparing mature, sclerotized individuals versus those partially melanized and sclerotized.

Features of the male aedeagal internal sac were described based on the homology system of Maddison (1993). Terms for the ovipositors used in the female genitalic descriptions were based on those presented by Ball and Shpeley (1983) and Liebherr and Will (1998). The number of male or female individuals dissected is noted in the appropriate section of the description.

## Taxonomic treatment

Moriomorphini Sloane, 1890: 646 (sensu Liebherr 2011a; type genus *Moriomorpha* Castelnau).

Subtribe Moriomorphina Sloane, 1890: 646.

Melisoderides Sloane, 1898: 470 (synonymy Bouchard et al. 2011; type genus *Melisodera* Westwood).

Subtribe Amblytelina Blackburn, 1892: 85 (type genus *Amblytelus* Erichson).

Meonides Sloane, 1898: 470 (synonymy Liebherr 2011b; type genus *Meonis* Castelnau).

Tropopterides Sloane, 1898: 470 (synonymy Liebherr 2011b; type genus *Tropopterus* Solier).

Mecyclothoracitae Jeannel, 1940: 97 (synonymy Liebherr 2011b, type genus *Mecyclothorax* Sharp).

*Mecyclothorax* Sharp was classified in the subtribe Amblytelina based on the shared presence of elongate, apically narrowed male parameres that bear setae along the ventral margin of the right paramere and at the apex of the left paramere (Liebherr 2011b, fig. 6). The degree of parameral elongation and apical narrowing varies across the taxa placed in Amblytelina by that cladistic parsimony analysis, but all members of Amblytelina differ from those assigned to subtribe Moriomorphina, wherein the male parameres are parallel-sided, broad to their broadly rounded apex, and glabrous (e.g. Liebherr 2011b, fig. 1A–B). Within Amblytelina, *Amblytelus* Erichson and associated genera (Baehr 2004) comprise the sister group to *Mecyclothorax*. Taxa across Amblytelina exhibit derived transformations of the spermathecal duct entrance into the bursa, from the plesiomorphic position near the juncture of the common oviduct and bursa, to positions remote from that juncture. For example in *Amblytelus curtus* (F.) the spermathecal duct joins the bursa on its ventrolateral margin, about halfway from the bursal-oviduct juncture toward the bursal apex (Liebherr 2011b, fig. 3C). In many *Mecyclothorax* spp., the spermathecal duct-bursal juncture has transformed to a position on the dorsal surface of the bursa, directly dorsad the bursal-oviduct juncture (as in Fig. 5). However, in females of *Mecyclothorax lophoides* Chaudoir of Australia the spermathecal duct enters the bursa at the plesiomorphic position at the bursal-oviduct juncture; a plesiomorphic condition also exhibited by *Meonochilus bellorum* Liebherr of New Zealand (Liebherr 2011b, fig. 8A). With regard to the Society Islands’ *Mecyclothorax* fauna, the female reproductive tracts exhibit a spermathecal duct that enters the dorsal surface of the bursa copulatrix (Fig. 5), and male aedeagal internal sacs with a broadly rounded, spoon-shaped flagellar plate (Fig. 4G), both configurations shared with the Hawaiian generotype *Mecyclothorax montivagus* (Blackburn). These characters therefore support Perrault’s (1978a, b) generic assignment of the Society Islands’ fauna.

**Figure 2.**
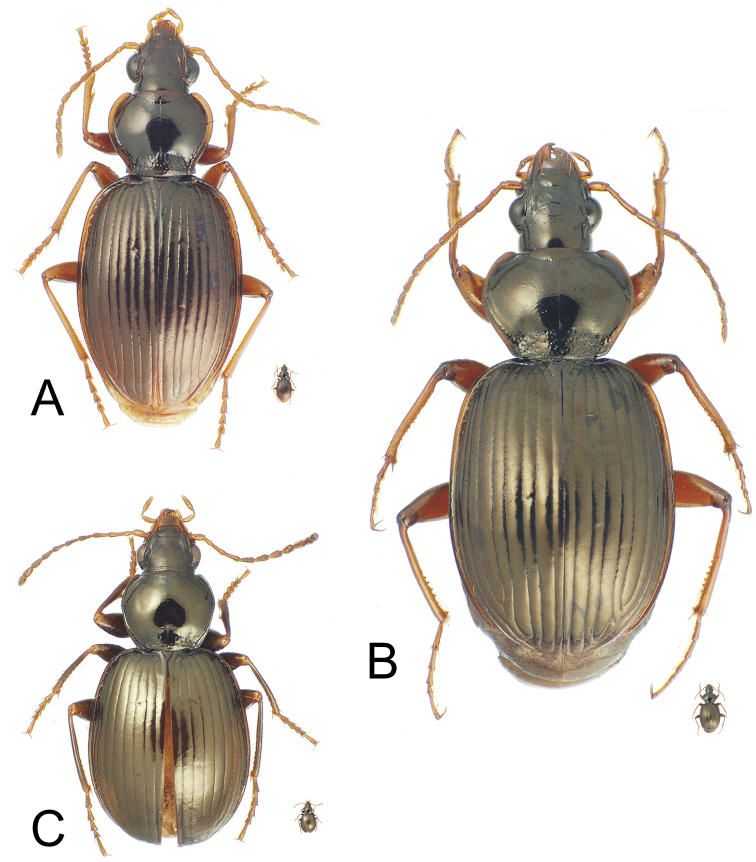
*Mecyclothorax* spp., dorsal view; silhouette to lower right of each habitus photo indicates actual size of beetle specimen at printed journal page size **A** *Mecyclothorax perraulti* male holotype **B** *Mecyclothorax pahere* male holotype **C** *Mecyclothorax menemene* male holotype.

**Figure 3.**
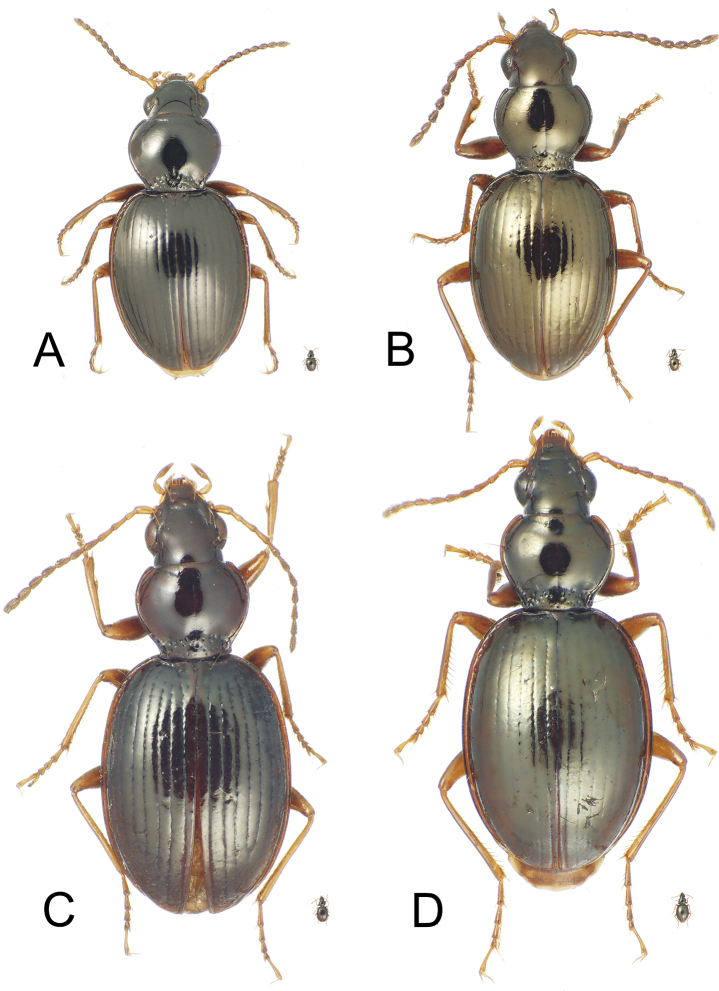
*Mecyclothorax* spp., dorsal view; silhouette to lower right of each habitus photo indicates actual size of beetle specimen at printed journal page size **A** *Mecyclothorax mahatahi* female holotype **B** *Mecyclothorax popotioaoa* male holotype **C** *Mecyclothorax mapo* male paratype (CUIC) **D** *Mecyclothorax fatata* female paratype (CUIC).

### Identification key to adults of *Mecyclothorax* spp. known from Moorea

**Table:** 

1	Pronotal lateral margin explanate throughout pronotal length, translucent	2
–	Pronotal lateral margin narrowly reflexed, edge narrowly upturned or beaded adjacent to pronotal lateral seta	3
2	Pronotal margin broadly explanate entire pronotal length, sinuate anterad hind angle	*Mecyclothorax perraulti* sp. n.
–	Pronotal margin broadest near posterior angle, narrowed anterad toward position of lateral seta, not sinuate anterad the broadly rounded hind angle	*Mecyclothorax pahere* sp. n.
3	Pronotum cordate, margin distinctly sinuate anterad hind angle	4
–	Pronotum ovate, margin straight to slightly convex anterad obtuse hind angle	*Mecyclothorax menemene* sp. n.
4	Both anterior and posterior supraorbital setae present, a thin carina present between dorsoanterior margin of eye and anterior seta	5
–	Posterior supraorbital seta present, anterior supraorbital seta absent, only a low broad convexity mesad dorsoanterior margin of eye	*Mecyclothorax mahatahi* sp. n.
5	Pronotum bisetose, both lateral and basal setae present; pronotum moderately cordate, MPW/BPW ratio 1.52–1.64, pronotal lateral margins subparallel to slightly divergent anterad obtuse-rounded hind angles	6
–	Pronotum unisetose, only the lateral seta present, hind angle glabrous; pronotum distinctly cordate, MPW/BPW ratio 1.67– 1.76, pronotal lateral margins convergent for ~¹�₉ pronotal length anterad sharply right hind angle	*Mecyclothorax popotioaoa* sp. n.
6	Body size larger, standardized body length 4.7–5.0 mm; a single dorsal elytral seta present at ~0.25 distance from base of scutellum to elytral apex; both apical and subapical elytral setae present	*Mecyclothorax mapo* sp. n.
–	Body size smaller, standardized body length 3.8–4.4 mm; two dorsal elytral setae positioned at ~0.32–0.34 and ~0.66–0.68 distance from base of scutellum to elytral apex; apical elytral seta (apex 2^nd^ stria) present, subapical elytral seta (in 7^th^ stria) absent	*Mecyclothorax fatata* sp. n.

### *Mecyclothorax gourvesi* species group

**Diagnosis.** Perrault (1986, 1988) based recognition of this species group on presence of a broadly margined pronotum, with sinuate basolateral margins and glabrous, right hind angles.

#### Mecyclothorax perraulti sp. n.

urn:lsid:zoobank.org:act:F7C6AC8A-46B8-4E9C-9B58-645A839318C8

http://species-id.net/wiki/Mecyclothorax_perraulti

##### Diagnosis.

**Figure 4.**
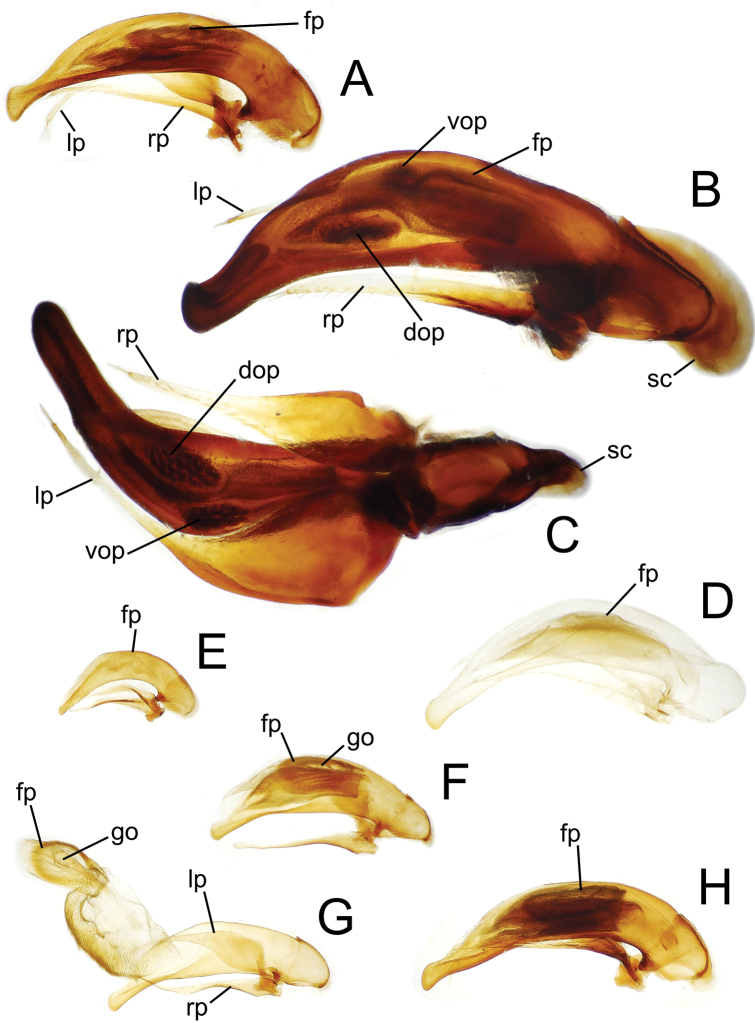
Male aedeagal median lobe and associated parameres. **A** *Mecyclothorax perraulti*, right lateral view **B** *Mecyclothorax pahere*, right lateral vew **C** *Mecyclothorax pahere*, euventral view **D** *Mecyclothorax menemene*, right lateral view (teneral specimen) **E** *Mecyclothorax popotioaoa* , right lateral view **F** *Mecyclothorax mapo*, right lateral view **G** *Mecyclothorax mapo*, right lateral view, internal sac everted **H** *Mecyclothorax fatata*, right lateral view. Abbreviations: **dop** dorsal ostial microtrichial patch **fp** flagellar plate **go** gonopore **lp** left paramere **rp** right paramere **sc** sagittal crest **vop** ventral ostial microtrichial patch.

Consistent with species group membership, the pronotal lateral margins are broadly explanate and translucent, with the basolateral margin sinuate anterad the projected, nearly right hind angles (Fig. 2A). The elytral lateral margin is also broadly expanded and translucent, with the margin upraised just laterad the angulate humerus. *Mecyclothorax perraulti* is most similar to *Mecyclothorax gourvesi*, though the pronotal base is narrower relative to pronotal maximum width in *Mecyclothorax perraulti* (MPW/BPW = 1.49–1.52), versus the basally broader pronotum of *Mecyclothorax gourvesi* (MPW/BPW = 1.37–1.39). The male aedeagal median lobe of *Mecyclothorax perraulti* has a broadened, adze-shaped apex, with the apical face straighter than the dorsal and ventral margins (Fig. 4A). Individuals of *Mecyclothorax perraulti* are also larger than those of *Mecyclothorax grouvesi*; standardized body length 5.8–6.2 mm versus a body length of 5.5 mm for the latter (Perrault 1988). Setal formula: 2122.

##### Description.

*Head capsule* with broadly excavate, straight frontal grooves, bordered laterally by a finely raised carina just mesad the anterior supraorbital seta, the mesal face indistinctly wrinkled between the eyes; neck not depressed dorsally between the eyes, frons flat in lateral view; ocular lobe distinctly projected, the posterior margin abruptly meeting gena behind eye, a fine, shallow groove at ocular lobe-genal juncture; ocular ratio 1.48–1.58 (n = 4); ocular lobe ratio 0.79–0.85 (n = 4); anterior margin of labrum broadly, shallowly emarginate, emargination about ¹�₉ labral length; antennomeres 1–3 glabrous except for apical setae; antennae filiform, antennomere 8 length 2.4× greatest width; mentum tooth with sides defining an acute angle, the apex tightly rounded. *Prothorax* moderately transverse, MPW/PL = 1.24–1.31 (n = 4); median base slightly depressed relative to disc, 25–27 small, isolated punctures each side of depressed area; basal margin broadly convex between laterobasal depressions, nearly straight medially; median longitudinal impression fine and shallow on disc, a lenticular broadening at front of median base; anterior transverse impression finely incised throughout breadth, shallower medially; anterior callosity slightly convex, crossed with indistinct longitudinal wrinkles; front angles protruded, subangulate medially adjacent to head, broadly convex laterally; distance between front angles less than between hind angles, APW/BPW 0.87–0.94 (n = 4); lateral marginal depression canaliculate where the convex pronotal disc meets the explanate, angularly upraised lateral margin; laterobasal depression a linear extension of the narrow, deep lateral depression; proepisternum with ~7 indistinct punctulae along hind margin, smaller irregularities along posterior marginal bead of proepimeron; prosternal process broad, slightly depressed anterad coxal cavities, convex posteriorly at juncture with posterior face. *Elytra* subquadrate, MEW/HuW = 2.29–2.37 (n = 4); disc flat medially, markedly sloped laterad interval 7 to near vertical juncture with lateral marginal depression; basal groove indistinctly though broadly recurved to angulate humerus; parascutellar seta present; parascutellar striole 6–7 punctate, continuously depressed between punctures; sutural interval elevated to sutural juncture, more convex than intervals 2–4; sutural and second striae subequally depressed at elytral apex; striae 1–7 minutely punctate basally, smooth apically, intervals distinctly convex; eighth interval distinctly carinate laterad stria 7 in apical half of elytron, the lateral portions of interval 8 depressed enhancing carina and change in discal curvature; 2 dorsal elytral setae, positioned at 0.24–0.25× and 0.57–0.61× elytral length, each seta in an indistinct depression that spans half or less the width of interval 3; both apical and subapical setae present; lateral elytral setae 7 + 6; elytral marginal depression broadly reflexed, translucent posterolaterad humerus; slightly broader laterad anterior lateral elytral setal series, narrowed apically to a beadlike margin anterad subapical sinuation; subapical sinuation abrupt, internal plica evident beneath sinuation in ectal view. *Mesepisternum* distinctly punctate anteriorly, ~18 evident punctures in 2–3 rows; metepisternum moderately foreshortened, width to length ratio 0.67; mesepisternal/mesepimeral suture distinct; metathoracic flight wing a narrow, elongate vestigium, length 3× width, remnants of veins C, R, M, and Cu discernible. *Abdomen* with visible ventrites 1–4 irregularly wrinkled laterally, ventrites 3–6 with round depressions laterally; suture between visible ventrites 2 and 3 effaced laterally. *Legs* moderately elongate, ratio of metatarsomere 1 length to metatibia length 0.23; metatarsomere 4 lobate, length including outer apical lobe 1.5× median tarsomere length; metatarsomere 4 with apical setae only, subapical setae absent; metatarsal dorsolateral sulci very shallow, obsolete, tarsomere dorsum broadly convex. *Microsculpture* of frons obsolete, surface glossy, shallow isodiametric sculpticells in transverse rows on neck; pronotal disc with mesh of transverse sculpticells 2–3× broad as long; pronotal median base covered with irregular, swirling isodiametric and transverse sculpticells between punctures; elytral disc with transverse microsculpture, sculpticells connected into a mesh, to parallel, unconnected transverse lines; metasternum covered with distinct transverse mesh; basal 2 abdominal ventrites covered with swirling isodiametric and transverse sculpticells. *Coloration* a somber reddish brown; head capsule rufous with a piceous cast; antennomere 1 rufoflavous, 2–11 rufobrunneous; pronotal disc rufopiceous, base, apex and lateral margins rufobrunneous; proepipleuron rufoflavous, proepisternum rufous; elytral disc rufobrunneous with purplish reflection due to transverse microsculpture; sutural interval paler, rufous basally, rufoflavous apically; elytral lateral margins rufoflavous, lateral marginal depression flavous inside dark margin; elytral apex broadly, slightly paler, rufoflavous; elytral epipleuron rufoflavous, metepisternum rufobrunneous with a piceous cast; abdominal ventrites 1–3 rufobrunneous, 4–6 paler, rufoflavous; metafemur rufoflavous with medial brunneous cloud; metatibia rufoflavous with brunneous cast.

**Male Genitalia.** (n = 1). Aedeagal median lobe narrowed dorsoventrally in apical half, apex dorsoventrally expanded to an adze-like tip (Fig. 4A), the apical face of tip much less convex than dorsal and ventral margins; flagellar plate large, length 0.5× distance from parameral articulation to apical face of tip; right paramere extended to 0.8× distance from parameral articulation to apical face, left paramere extended nearly to tip; internal sac with broad dense ventral microtrichial field, and small, lightly spinose dorsal ostial microtrichial patch (assessed in uneverted condition).

**Figure 5.**
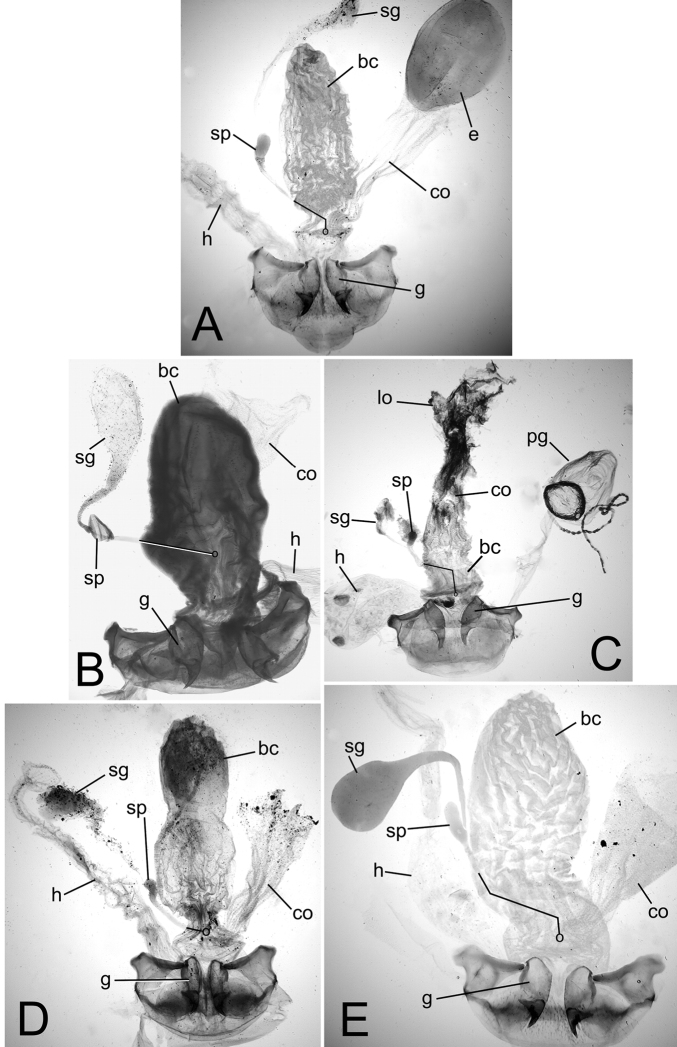
Female reproductive tract and associated abdominal structures, ventral view. Black line indicates position of spermathecal duct dorsad bursa copulatrix or common oviduct; circle at end of line indicates position of juncture of spermathecal duct and dorsal wall of bursa. **A** *Mecyclothorax perraulti* **B** *Mecyclothorax pahere* **C** *Mecyclothorax popotioaoa* **D** *Mecyclothorax mapo* **E** *Mecyclothorax fatata*. Abbreviations: **bc** bursa copulatrix **co** common oviduct **e** egg **g** gonocoxa **h** rectum of hindgut **lo** lateral oviduct **pg** pygidial gland reservoir **sg** spermathecal gland **sp** spermatheca.

**Figure 6.**
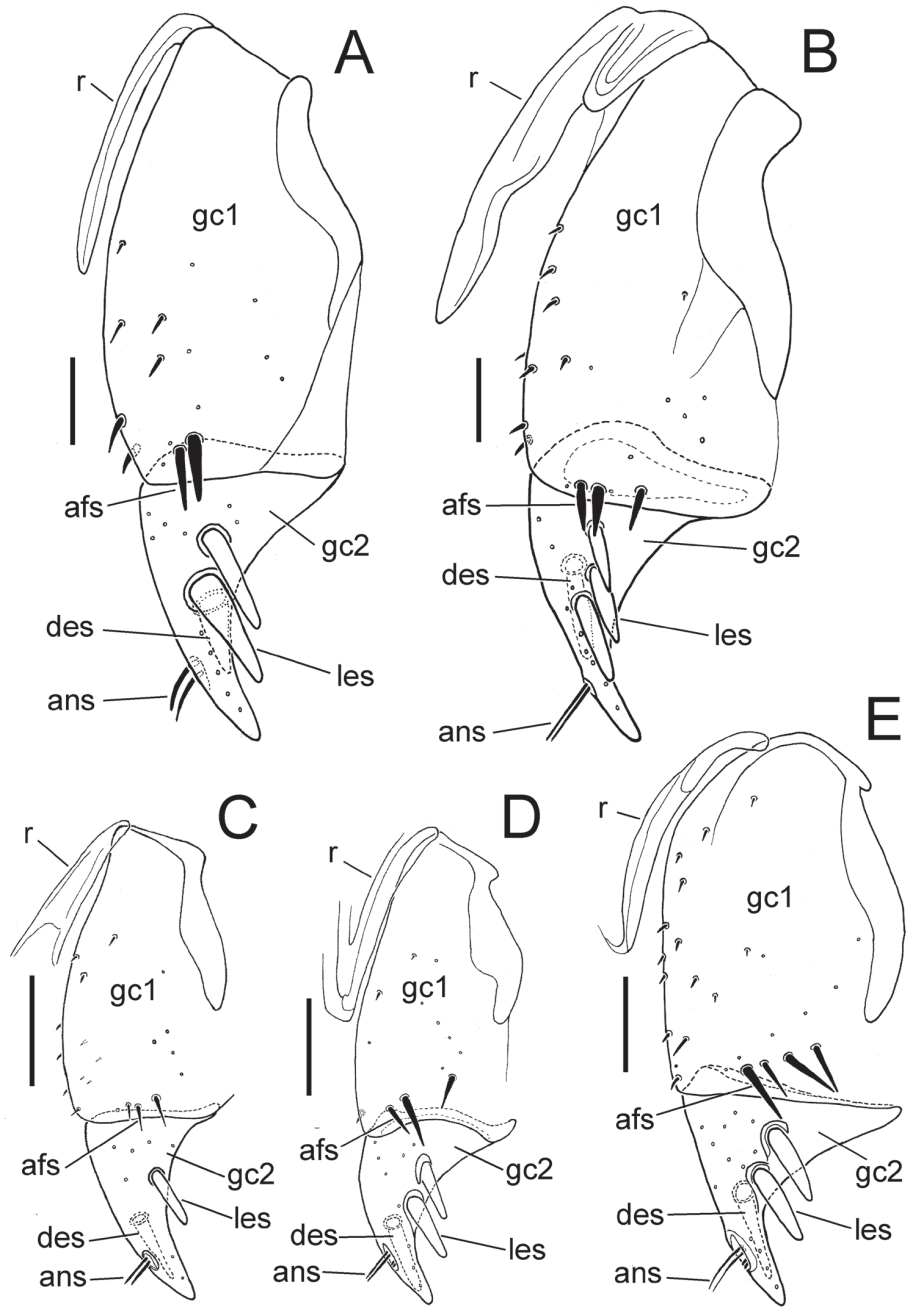
Female left gonocoxa, ventral view; scale bar = 0.05 mm. **A** M. *perraulti* **B** M. *pahere* **C** *Mecyclothorax popotioaoa* **D** *Mecyclothorax mapo* **E** *Mecyclothorax fatata*. Abbreviations: **afs** apical fringe setae of gonocoxite 1 **ans** apical nematiform seta **des** dorsal ensiform seta **gc1** basal gonocoxite 1 **gc2** apical gonocoxite 2 **les** lateral ensiform seta(e) **r** ramus.

**Female Reproductive Tract.** (n = 1). Bursa copulatrix columnar, elongate, length 3× maximum breadth in slide-mounted specimen (Fig. 5A); bursa membranous, lightly sclerotized based on amount of Chlorazol Black staining; spermatheca reniform, spermathecal gland duct long enough so that gland extends beyond apex of bursa; basal gonocoxite 1 with apical fringe of 2–3 setae, 6–7 smaller setae along mesal margin (Fig. 6A); apical gonocoxite 2 subacuminate, apex tightly rounded, basolateral area moderately expanded; 2 lateral ensiform setae, the apical seta stouter, 1 dorsal ensiform setae, and 2 apical nematiform setae.

**Holotype** male (MNHN), labeled: FRENCH POLYNESIA: Moorea / Toheia, off trail beneath ridge / 26-ix-2009 el. 1145 m C. Ewing/-17.55152 -149.82147 pyr. fog / unknown tree MBIO6551 // HOLOTYPE / Mecyclothorax / perraulti / J.K. Liebherr 2012 (black-bordered red label)

**Allotype** female (MNHN), labeled: FRENCH POLYNESIA / Moorea Tohiea summit / 12-IX-2006 lot 07 / S17°33.03', W149°49.33' / el. 1150-1200 m beating / flowering *Myrsine* after / dark J.K. Liebherr // ALLOTYPE / Mecyclothorax / perraulti / J.K. Liebherr 2012 (black-bordered red label)

##### Other paratypes.

SOCIETY ISLANDS. Moorea: Tohiea summit, 1120 m el., S17°33.07', W149°49.38', 12-ix-2006 lot 05, beating rotten *Freycinetia* and ferns, Liebherr (CUIC, 1); 1097 m el., S17°33.05', W149°49.28', 13-ix-2006 lot 02, Berlese extraction of fern litter, Ewing (CUIC, 1); muddy gulch on trail, 1170 m el., S17°33.08', W149°49.31', 25-ix-2009, pyrethrin fog mossy tree, MBIO 5853, Ewing (EMEC, 1).

##### Etymology. 

This species epithet honors the memory of Georges G. Perrault, the principal describer and reviser of the Tahitian *Mecyclothorax* fauna.

##### Distribution and habitat.

Individuals of this species have been found from the summit at 1207 m elevation down to 1100 m. Specimens have been found microsympatrically in rotten *Freycinetia* stalks with *Mecyclothorax mapo*, and in association with *Mecyclothorax mapo* and *Mecyclothorax fatata* on flowering *Myrsine* at night, and in a pyrethrin fog sample of a mossy tree trunk. An individual of this species was the lone carabid beetle recovered from a Berlese extraction sample of leaf litter taken at 1100 m.

### *Mecyclothorax altiusculus* species group

**Diagnosis.** All species of this group as first defined by Perrault (1986) lacked the basolateral pronotal seta, though in his grouping the pronotal shape varied from trapezoidal with a convex or straight basolateral margin, to cordate wherein the basolateral margin is sinuate anterad a nearly right hind angle. Subsequently Perrault (1988) placed three species with basolateral pronotal setae in the group, while surmising that the species group was composed of several phylogenetic elements that might bear subdivision. The two new species placed here have the glabrous pronotal hind angles and convex basolateral pronotal margins also exhibited by *Mecyclothorax altiusculus* Britton, suggesting that these new species would remain in this group after such a subdivision.

#### Mecyclothorax pahere sp. n.

urn:lsid:zoobank.org:act:52B4B915-D08B-4409-A118-4901879032DE

http://species-id.net/wiki/Mecyclothorax_pahere

##### Diagnosis.

This species plus *Mecyclothorax altiusculus*, *Mecyclothorax pseudaltiusculus* Perrault, and *Mecyclothorax paraltiusculus* Perrault share a bisetose, trapezoidal pronotum with rounded hind angles, and a setal formula of 2122, however individuals of this new species are larger; standardized body length 7.5–7.9 mm. The pronotum is also more transverse; MPW/PL = 1.35–1.36 (n = 2). The striae are deep and distinctly punctate in their basal half, and the convex intervals are covered with dense transverse microsculpture consisting of a mixture of parallel transverse lines and transverse-mesh sculpticells 2–4× broad as long. The mesal face of the male metatibia is lined with pectinate swellings at the points of articulation of the mesal longitudinal setal series (Fig. 2B). Of the three species listed above, *Mecyclothorax paraltiusculus* is most similar, attaining a similar body size—7.0 mm—and possessing elytral microsculpture consisting of a mixture of transverse lines and transverse mesh.

##### Description.

*Head capsule* withbroad, shallow frontal grooves, the frontal surface transversely wrinkled between the grooves, and a broad, low convexity bordering the groove mesad the anterior supraorbital seta; dorsally the head capsule is flat from the frons to the pronotum; ocular lobe moderately prominent and largely covered by eyes, the posterior portion of lobe obtusely joined to gena, the juncture marked by fine, shallow groove; ocular ratio 1.47, ocular lobe ratio 0.79–0.83; labral anterior margin broadly emarginate 0.2× length; antennomeres glabrous except for apical setae; antennae elongate filiform, antennomere 8 length 3× greatest width; mentum tooth with sides defining an acute angle, the apex rounded. *Prothorax* transverse, the basolateral margins straight to slightly concave due to the upcurved margin anterad the rounded hind angles, MPW/BPW = 1.55–1.60 (n = 2); median base slightly depressed medially, moreso laterally, with more than 40 small punctures each side; basal margin broadly, slightly convex between laterobasal depressions; median longitudinal impression obsolete but traceable on disc, present as a lenticular depression at front of median base; anterior transverse impression deep, finely incised, with 7–8 elongate punctures each side bordered by longitudinal carinae that span impression from disc to anterior callosity; anterior callosity moderately convex, covered with indistinct longitudinal wrinkles; front angles protruded, broadly rounded, APW/BPW = 0.95–0.96 (n = 2); lateral marginal depression broadly explanate, translucent, edge upturned anteriorly, more beadlike near lateral seta; laterobasal depression a broadened continuation of the lateral depression, surface punctured as median base, deepest portion meeting posterior margin at lateral edge of basal margin convexity; proepisternum with 7 distinct punctulae along hind margin, ~10 smaller punctures along posterior marginal bead of proepimeron; prosternal process broad, slightly depressed anteriorly between coxal cavities, convex posterad at juncture with posterior face. *Elytra* broad, subquadrate, MEW/HuW = 2.32–2.35 (n = 2); disc convex medially, sides sloped to nearly vertical; basal groove moderately curved to subangulate humerus, elytral margin at humerus only slightly upraised; parascutellar seta present; parascutellar striole defined by 4 separated punctures, the striole depressed between punctures; sutural intervals elevated to meet at suture, only slightly more convex than distinctly convex discal intervals; striae 1–8 deep, finely impressed, and distinctly punctate in basal half; all striae deep and smooth at elytral apex; eighth interval upraised in a narrow bulbous carina laterad subapical seta, the interval’s outer face nearly vertical; 2 dorsal elytral setae (one individual with 3 setae on one elytron) positioned at 0.22× and 0.52–0.60× elytral length (asymmetrical third seta at 0.75× elytral length), each seta within evident depressions that span ⅔ width of interval 3; both apical and subapical elytral setae present; lateral elytral setae 7 + (5–6); elytral marginal depression moderately broad throughout length, translucent, reduced to beadlike margin only just anterad abruptly concave subapical sinuation. *Mesepisternum* distinctly punctate anteriorly, ~19 deep punctures in 2–3 rows; metepisternum short, width to length ratio 0.87; metepisternal-metepimeral suture varied, a distinct suture in one individual, an indistinct, broad depression in the second; metathoracic flight wing an elongate straplike vestigium, length 4× width, and apical ¼ of wing length surpassing hind margin of metathorax, rudimentary R and M veins evident. *Abdomen* with irregular wrinkles on visible ventrites 1–4, and indistinct round depressions laterally on ventrites 4–6; suture between visible ventrites 2 and 3 complete. *Legs* with metatarsomere 1 moderate, length 0.19× length of metatibia; metatarsomere 4 with short apical lobes, maximal tarsomere length 1.2× median tarsomere length; metatarsomere 4 with very short subapical setae and longer apical setae; metatarsal dorsolateral sulci very shallow, obsolete, dorsum broadly convex. *Microsculpture* on frons and vertex a transverse mesh, sculpticell breadth 2× length; pronotal disc with obsolete microsculpture, indistinct transverse mesh with sculpticells 3–4× broad as long visible near edge of light reflections; pronotal median base with evident transverse-mesh microsculpture between punctures, sculpticell breadth 2–3× length; elytral disc covered with a mixture of transverse lines and transverse mesh with sculpticell breadth 2–4× length; elytral apex covered with transverse mesh, sculpticell breadth 2–4× length; metasternum glossy with obsolete transverse sculpticells; laterobasal abdominal ventrites glossy with shallow, swirling isodiametric and transverse mesh sculpticells. *Coloration* of frons and vertex piceous; antennomeres 1–4 rufoflavous to brunneous, segments 5–11 slightly darker; pronotal disc rufopiceous with metallic silvery reflection; reflexed pronotal margins translucent brunneous; proepipleuron rufo-brunneous, proepisternum rufopiceous; elytral disc rufopiceous with cupreous reflection; sutural interval concolorous basally, rufous apically; reflexed elytral margin brunneous, elytral apex narrowly, indistinctly paler, brunneous; elytral epipleuron rufobrunneous, metepisternum rufopiceous; abdominal ventrites glossy rufopiceous, apical ventrite narrowly rufobrunneous; metafemur and metatibia rufobrunneous.

**Male genitalia.** (n = 1). Aedeagal median lobe broadest dorsoventrally near midlength, apex hooklike, with small dorsal toothlike expansion (Fig. 4B); median lobe curved to right in ventral view (Fig. 4C), the apex elongated beyond apical margin of ostium; internal sac with well-developed dorsal and ventral ostial microtrichial patches; flagellar plate moderately large, length 0.3× distance from apex to parameral articulation; parameres broad basally, right paramere extended 0.7× distance from parameral articulation to apex (Fig. 4B), left paramere longer, extended 0.9× distance to apex (Fig. 4C).

**Female reproductive tract.** (n = 1). Bursa copulatrix columnar, heavily sclerotized, the surface leathery, bursal length 2× maximal width in slide-mounted dissection (Fig. 5B); spermathecal duct entering on dorsal bursal wall apicad position along length of bursal juncture with common oviduct; spermathecal gland duct short, little longer than spermatheca, the glandular reservoir elongate and gradually widened in diameter from duct; basal gonocoxite 1 with 3–4 apical fringe setae, and 8–10 setae along ventromesal margin (Fig. 6B); apical gonocoxite 2 broad basally with narrow, acuminate tip, and 2–3 lateral ensiform setae, 1 dorsal ensiform seta, and 2 apical nematiform setae.

**Holotype** male (MNHN), labeled: FRENCH POLYNESIA: Moorea / Tohiea, muddy gulch along trail / 24-ix-2009 el. 1150 m C. Ewing / -17.55130 -149.82178 pyr. fog / mossy tree trunk MBIO 5860 // HOLOTYPE / Mecyclothorax / pahere / J.K. Liebherr 2012 (black-bordered red label).

**Allotype** female (MNHN), labeled: FRENCH POLYNESIA: Moorea / Tohiea, muddy gulch along trail / 25-ix-2009 el. 1150 m C. Ewing / -17.55130 -149.82178 pyr. fog / mossy tree trunk MBIO 5859 // ALLOTYPE / Mecyclothorax / pahere / J.K. Liebherr 2012 (black-bordered red label).

##### Etymology.

The species epithet is the Tahitian word pahere, or comb in English, either the noun or verb form (Wahlroos, 2002), and being indeclinable, is to be treated as a noun in apposition. The name is indicative of the metatibial comb in the male, formed by the evaginated bosslike articulatory processes associated with with the mediolongitudinal series of tibial setae.

**Distribution and habitat.** The allotype female was collected in a pyrethrin fog sample from a mossy tree trunk along with one individual each of *Mecyclothorax fatata* and *Mecyclothorax mapo*. The holotype male comprised the only beetle collected in a similar situation the day earlier.

#### Mecyclothorax menemene sp. n.

urn:lsid:zoobank.org:act:2D31BAF6-A9E3-4344-BE4D-32658F0D209E

http://species-id.net/wiki/Mecyclothorax_menemene

##### Diagnosis.

This species shares an ovoid, bisetose pronotum, and indistinctly striate elytra with *Mecyclothorax jarrigei* Perrault, however the striae are even less developed in *Mecyclothorax jarrigei*, and individuals of that species at 6.2 mm are larger than the unique specimen of this new species; standardized body length 5.2 mm. This species exhibits a setal formula of 2121 as in *Mecyclothorax jarrigei*; setae in the new species include both discal elytral setae and the apical elytral seta positioned just laterad the apex of the second stria. The elytra are exceedingly convex in the unique holotype (Fig. 2C), perhaps due to the slightly teneral nature of the specimen leading to apical distortion of the elytra. The dense transverse microsculpture on the elytra results in a silvery metallic reflection.

##### Description.

*Head capsule* frontal grooves smooth, with deepest portions sinuously curved mesad anterior margin of eye, broadest near frontoclypeal suture, and with low rounded carina mesad anterior supraorbital seta; dorsum of head slightly concave between anterior portions of eyes, neck slightly convex; ocular lobe broadly protruded, the posterior portion very obtusely meeting gena at very shallow ot obsolete groove; eyes small, about 16 ommatidia along a horizontal diameter commencing posterad ventral edge of antennal articulatory socket; ocular ratio 1.36, ocular lobe ratio 0.75; labral anterior margin straight; antennomeres 1–3 glabrous except for apical setae; antennae robust, submoniliform, antennomere 8 length 1.9× greatest width; mentum tooth with sides defining acute angle, apex rounded. *Prothorax* subovoid, hind angle definable by abrupt change in curvature of pronotal margin, but basolateral margin convex anterad angle, MPW/PL = 1.17 (n = 1); median base slightly depressed relative to disc, ~10 punctures of varying depth each side; basal margin slightly convex between laterobasal depressions; median longitudinal impression obsolete on disc, indicated by darker color of endocarina, present as a narrow elongate depression on front of median base; anterior transverse impression very fine and shallow, obsolete medially, finely incised mesad front angles; anterior callosity narrow, slightly elevated but flat, crossed by irregular longitudinal wrinkles; front angles slightly protruded, rounded, distance between somewhat greater than basal pronotal width, APW/BPW = 1.08; lateral marginal depression very narrow laterally, margin beaded, slightly broader with edge little upturned at front angles, gradually broadened toward laterobasal depression, edge upturned basally; laterobasal depression a broadened continuation of lateral depression, deepest portions mostly smooth but with minute irregularities; proepisternum with ~7 indistinct punctulae along hind margin, proepimeron with minute irregularities along marginal collar; prosternal process broad, slightly depressed mesad anterior margins of procoxal cavities, convex posterad at juncture with posterior face. *Elytra* subovoid, disc upraised above position of scutellum, MEW/HuW = 2.39; basal groove moderately curved to meet subangulate humerus; parascutellar seta present; parascutellar striole shallow, broadly impressed, smooth; sutural interval slightly elevated at sutural juncture, but not more convex than interval 2 laterally; striae 1–8 of same depth in basal half, shallow but complete, generally smooth but with minute irregularities associated with slight change of direction of deepest portions; at elytral apex, sutural striae 1 and 7 deeply incised, stria 2 shallow, almost discontinuous, striae 3 and 4 shallow but continuous; eighth interval convex apically, the convexity oriented laterally above subapical sinuation; 2 dorsal elytral setae at 0.25× and 0.60× elytral length, set in a moderate impression that spans ⅔ width of interval 3; apical elytral seta present, subapical seta absent; lateral elytral setae 7 + 6; elytral marginal depression narrow but margin narrowly upturned at humerus, depression somewhat broader with more gently upturned edge at midlength, margin becoming less upturned without bead anterad subapical sinuation; subapical sinuation shallow, only slightly changing curvature of apical marginal bead. *Mesepisternum* punctate anteriorly, ~18 punctures in 2–3 rows; mesepisternum longer than broad, width to length ratio 0.77; metepisternum separated from metepimeron by distinct suture; metathoracic flight wing vestigium apex extended to hind margin of metathorax, venation not assessed. *Abdomen* with irregular wrinkles laterally on visible ventrites 1–4, indistinct rounded impressions laterally on ventrites 4–6; suture between ventrites 2 and 3 complete laterally. *Metatarsomere 4* with emarginate apical margin, overall length including apical lobes 1.4× median tarsomere length; metatarsomere 4 with short subapical and longer apical setae; metatarsal dorsolateral sulci shallow, lateral, the median surface broadly convex; male metatibia with four rounded projections at bases of four apical setae in mesal longitudinal series. *Microsculpture* of frons, obsolete, surface glossy, neck with indistinct isodiametric sculpticells in transverse rows; pronotal disc covered with shallow transverse mesh, sculpticell breadth 2–4× length; pronotal median base with shallow, swirling isodiametric and transverse sculpticells between punctures; elytral disc covered with mixture of well-developed transverse mesh, sculpticell breadth 2–4× length, and transverse lines; eytral apex with transverse mesh, sculpticells 2–4× broad as long; metasternum with distinct transverse-mesh microsculpture; laterobasal abdominal ventrites covered with swirling isodiametric and transverse mesh. *Coloration* of head capsule rufous with piceous cast; antennomeres 1–2 rufoflavous, 3–11 darker, brunneous; pronotal disc rufobrunneous medially, darker than head laterally on disc, lateral marginal depression concolorous, only extreme basal and apical margins paler, rufoflavous; proepipleuron dark rufoflavous, proepisternum rufobrunneous; elytral disc rufobrunneous with silvery reflection, sutural interval concolorous; elytral marginal depression narrowly translucent, slightly paler than disc, apex not paler than more basal portions; elytral epipleuron, metepisternum, and abdominal ventrites concolorous, rufobrunneous; metafemur and metatibia brunneous medially, apices paler, rufoflavous.

**Male** **Genitalia.** (n = 1). Aedeagal median lobe evenly narrowed dorsoventrally from midlength to broadly rounded apex, the apex slightly expanded on ventral margin (Fig. 4D); flagellar plate smaller, length 0.26× distance from parameral articulation to apex (assessed in uneverted, teneral dissection); internal sac dorsal surface apparently covered with field of fine spicules, neither dorsal nor ventral ostial microtrichial patch apparent; both parameres extended apically about 0.8× distance from parameral articulation to apex.

**Holotype** male (MNHN), labeled: French Polynesia: Moorea / Tohiea summit el. 1130 m / 12-IX-2006 lot10 / 17°33.07'S, 149°49.38'W / pyrethrin fog mossy logs / D.A. Polhemus// HOLOTYPE / Mecyclothorax / menemene / J.K. Liebherr 2012.

##### Etymology.

The species epithet is the Tahitian word menemene, i.e. round or spherical in English (Wahlroos, 2002), denoting the rounded pronotum and broadly rounded, convex elytra of this species. Being indeclinable, the epithet is to be treated as a noun in apposition.

##### Distribution and habitat.

The unique holotype of this species was collected from a rotten log situated in a deep, wet gulch, in association with four individuals of *Mecyclothorax mapo* and one of *Mecyclothorax popotioaoa*.

### *Mecyclothorax globosus* species group

**Diagnosis.** Species in this group are characterized by cordate pronota, the basolateral margin distinctly sinuate anterad the projected hind angle, with the lateral marginal depression very narrow and of even breadth throughout the length of the pronotum. The elytra are very convex, with the eighth interval nearly vertical at the juncture with the elytral lateral marginal depression (Perrault, 1986, 1988). Individuals of the included species are smaller; body lengths range 3.3–5.0 mm (Perrault, 1989).

#### Mecyclothorax mahatahi sp. n.

urn:lsid:zoobank.org:act:A5DBBF5A-AAF8-487C-90B1-20CDD9E34633

http://species-id.net/wiki/Mecyclothorax_mahatahi

##### Diagnosis.

This species shares reduced setation, setal formula 1111 (Fig. 3A), with four other species in the group; *Mecyclothorax sabulicola* Britton, *Mecyclothorax ataraensis* Perrault, *Mecyclothorax taiarapu* Perrault, and *Mecyclothorax cupripennis* Perrault. Of these, *Mecyclothorax cupripennis* shares reduced microsculpture with this species, the pronotal disc lacking any discernible sculpticells. The two species differ in elytral microsculpture. This new species is characterized by a glossy elytral integument, with only sporadic small patches of indistinct isodiametric sculpticells in transverse rows, whereas *Mecyclothorax cupripennis* is characterized by presence of a more regular, though shallow, transverse mesh on the discal elytral intervals; the sculpticells consistently visible outside the reflection of bright, direct microscope light. Body size is similar for the two species; standardized body length of the new species is 3.7 mm, that for *Mecyclothorax cupripennis* 3.5 mm (measurement made on male specimen, CUIC).

##### Description.

*Head capsule* withsinuous frontal grooves, closest at frontoclypeal suture and defining a lyre shape, adjacent area of frons broadly depressed mesad anterior margin of eye, base of frontal groove separated from eye by a broad, low convexity; dorsum of head flat on frons in lateral view, neck convex; anterior supraorbital seta absent, posterior seta situated at dorsal terminus of broad shallow groove between ocular lobe and gena; eyes and ocular lobe little protruded, posterior portion of lobe meeting gena at about 135˚ angle; compound eye with 10 ommatidia on horizontal diameter defined by lower margin of antennal articulatory socket; ocular ratio 1.37, ocular lobe ratio 0.67; labral anterior margin broadly, shallowly emarginate ¹⁄₁₂ length; antennomeres 1–3 glabrous escept for apical setae; antennae moniliform, antennomere 8 length subequal to greatest width; mentum tooth with sides defining acute angle, apex tightly rounded. *Prothorax* cordate, disc convex, basolateral margins convergent anterad acute, projected hind angles, MPW/BPW = 1.53, MPW/PL = 1.18 (Fig. 3A); median base slightly depressed relative to disc, moreso laterally, with ~13 larger isolated punctures each side; basal margin broadly convex between laterobasal depressions; median longitudinal impression absent on basal half of disc, obsolete and traceable anterad due to indistinct transverse wrinkles at position of impression; anterior transverse impression shallow, broad, smooth medially, finely incised in lateral ⅔ of breadth; anterior callosity slightly convex, smooth; front angles slightly protruded, tightly rounded, APW/BPW = 0.99 (n = 1); lateral marginal depression very narrow, slightly broader at front angle, edge beaded throughout; laterobasal depression a deep continuation of lateral depression, bordered anteromesally by punctate median base, and laterally and posteriorly by raised marginal bead at hind angle; proepisternum with 5 minute punctures along hind margin; prosternal process narrowly depressed medially, broadly upraised each side between coxae. *Elytra* subovoid, MEW/HuW = 2.14 (n = 1), middle of disc flat, intervals 2–8 increasingly depressed to near vertical juncture with lateral marginal depression; basal groove distinctly curved forward to angulate humerus that lies distinctly anterad base of scutellum; parascutellar seta present, immediately adjacent to parascutellar striole; parascutellar striole finely incised, smooth anterad, 1–2 small punctures near apex; sutural interval coplanar with stria 2; striae 1–6 shallow, complete, with very small punctures at strial depth, the punctures less distinct in lateral striae; sutural stria 1 deepest at elytral apex, striae 2, 3, and 7 shallow but complete apically; discal elytral intervals slightly convex; interval 8 narrowly subcarinate laterad apex of stria 7, slightly more convex than more mesal intervals; a single dorsal elytral seta at 0.24× elytral length set in small setal depression spanning less than half of interval 3; apical elytral seta present, subapical seta absent; lateral elytral setae 7 + 6; elytral marginal depression moderately narrow, margin upraised near humerus, beadlike only near subapical sinuation; subapical sinuation deep, abruptly excavate anteriorly. *Mesepisternum* punctate anteriorly, with ~14 punctures in 2–3 rows; metepisternum short, anterior and mesal edges subequal, width to length ratio 0.8; metepisternum separated from metepimeron by a distinct suture. *Abdomen* irregularly wrinkled on lateral portions of visible ventrites 1–4, indistinct rounded depressions laterally on ventrites 4–6; suture between ventrites 2 and 3 effaced laterally. *Legs* with short, stout tarsomeres, metatarsomere 4 overall length subequal to breadth, length including apical lobes 1.2× median tarsomere length; metatarsomere 4 with both apical and subapical setae; metatarsal dorsolateral sulci deep, lateral, median broadly convex. *Microsculpture* of frons obsolete, surface glossy, indistinct transverse mesh in deepest portions of frontal grooves; pronotal disc glossy with indistinct elongate transverse mesh—sculpticell breadth 3–4× length—visible near edge of areas of reflected light; pronotal median base glossy except for obsolete transverse mesh near discal margin; elytral disc mostly glossy, patches of transverse mesh, breadth 3–4× length, visible near striae; elytral apex glossy, transverse mesh visible at apical margin; metasternum with evident transverse mesh; laterobasal abdominal ventrites with swirling isodiametric and transverse sculpticells. *Coloration* of head capsule a glossy rufopiceous; antennomere 1 flavous, 2–3 rufoflavous, 4–11 rufobrunneous; pronotal disc glossy rufopiceous, pronotal lateral margins concolorous, base and apex slightly paler at edge; proepipleuron rufopiceous along edge, mediolongitudinally rufoflavous, rufobrunneous along margin with rufobrunneous proepisternum; elytral disc glossy rufopiceous; scutellum and base of sutural interval dark rufous, apex of sutural interval broadly rufobrunneous; elytral margin concolorous with disc near elytral base, lateral marginal depression paler, rufoflavous behind; elytral apex broadly, slightly paler, brunneous; elytral epipleuron paler, rufoflavous dorsally, rufobrunneous ventrally to match metepisternum; abdominal visible ventrite 1 rufobrunneous; ventrites 2–3 rufopiceous medially, brunneous laterally along with ventrites 4–6; apical ventrite 6 rufoflavous in apical ⅓; metafemur rufoflavous; metatibia rufoflavous with brunneous cast.

**Female reproductive tract.** The unique female holotype was not dissected.

**Holotype** female (MNHN), labeled: FRENCH POLYNESIA: Moorea / Tohiea, off trail beneath ridge / 25-ix-2009 el. 1145 m C. Ewing / -17.55152 -149.82147 pyr. fog / unknown tree MBIO5551 // HOLOTYPE / Mecyclothorax / mahatahi / J.K. Liebherr 2012 (black-bordered red label).

##### Etymology.

The species epithet is a compounding of maha, Tahitian for the number four, and tahi, Tahitian for one (Wahlroos, 2002), indicative of the reduced setation in this species resulting in the setal formula of 1111. As tahi is indeclinable, the epithet is to be treated as a noun in apposition.

##### Distribution and habitat.

The unique specimen was collected in pyrethrin fog sample of a mossmat in association with one specimen of *Mecyclothorax perraulti*.

#### Mecyclothorax popotioaoa sp. n.

urn:lsid:zoobank.org:act:CD0E0395-531F-4DD2-B68C-8761EA70D2F5

http://species-id.net/wiki/Mecyclothorax_popotioaoa

##### Diagnosis. 

Within the *Mecyclothorax globosus* group, this is the only species for which individuals lack dorsal elytral setae, resulting in a setal formula of 2101; one individual of the five type specimens has an asymmetrically positioned dorsal elytral seta at 0.24× length on the left elytron, however this is considered a variant condition not characterizing the species. This species is also characterized by shallow elytral striae (Fig. 3B), and much reduced microsculpture across the entire dorsum. The most similar species in the group, *Mecyclothorax hemisphaericus* Perrault, shares much reduced elytral striae, though in this species they are nearly obsolete. *Mecyclothorax hemisphaericus* also exhibits reduced setation, though presence of a single dorsal elytral seta results in a setal formula of 2111. The new species can also be distinguished from *Mecyclothorax hemisphaericus* by the more narrowly ovoid elytra, MEW/MPW = 1.43–1.48 (n = 4), versus more broadly ovoid elytra in *Mecyclothorax hemisphaericus*, MEW/MPW = 1.61 (n = 2 paratypes, MNHN). Individuals of both species are of similar size; standardized body length for this species is 3.7–4.0 mm, versus 3.5–3.9 mm for *Mecyclothorax hemisphaericus*, as determined from two examined paratypes (MNHN).

##### Description.

*Head capsule* withslightly sinuous frontal grooves, the two grooves approaching each other mesad a broad convexity near frontoclypeal suture, frons mesad grooves depressed and transversely wrinkled, groove bordered laterally by thin carina mesad anterior supraorbital seta; dorsum of head flat on frons in lateral view, neck convex; ocular lobe little projected, posterior portion meeting gena at >135˚, a narrow, shallow groove at juncture; eyes slightly more convex than ocular lobe, 14–15 ommatidia along horizontal diameter oriented to ventral margin of antennal articulatory socket; ocular ratio 1.36–1.42 (n = 4), ocular lobe ratio 0.74–0.76 (n = 4); labral anterior margin broadly, shallowly emarginate ¹�₆ of length; antennomeres 1–3 glabrous except for apical setae; antennae submoniliform, antennomere 8 length 1.75× greatest breadth; mentum tooth with sides defining an acute angle, apex tightly rounded. *Pronotum* narrow, little transverse, distinctly cordate, the basolateral margins subparallel to slightly convergent for ¹�₉ pronotal length anterad projected, slightly obtuse hind angles (Fig. 3B), MPW/BPW = 1.67–1.76 (n = 4), MPW/PL = 1.12–1.16 (n = 4); median base slightly depressed relative to disc, 12–14 large punctures each side; basal margin broadly convex between hind angles; median longitudinal impression very shallow, finely incised, but traceable across disc, briefly extended as an elongate puncture at front of median base; anterior transverse impression very shallow, smooth, obsolete medially, finely incised in outer half of breadth each side; anterior callosity slightly convex, smooth but with very minute longitudinal wrinkles; front angles not protruded anterad, the margin perpendicular to longitudinal axis and curving posterad in a tight curve, APW/BPW = 1.13–1.19 (n = 4); lateral marginal depression very narrow, pronotal margin beaded throughout length to hind angle and posterad laterobasal depression; laterobasal depressions ill defined, punctate, coplanar with lateral portion of median base; proepisternum with ~10 very fine irregularities along hind margin; prosternal process narrowly depressed medially, sides broadly upraised mesad coxal cavities. *Elytra* narrowly subovoid (Fig. 3B), disc convex, sides distinctly sloped to near vertical; basal groove evenly curved anterad to angulate humerus, MEW/HuW = 2.2–2.3 (n = 4); parascutellar seta present; parascutellar striole 3–4 punctate, shallow but continuous; sutural interval slightly elevated at sutural juncture basally, moreso apically; striae 1–6 shallow on disc, indistinctly punctate, stria 7 obsolete but traceable, smooth; striae 1 and 7 deep and smooth apically, striae 2–6 shallower but traceable, though juncture of 5 and 6 is deeper; discal intervals slightly convex, lateral intervals less upraised, though convex due to curvature of elytron; interval 8 distinctly bulbous laterad stria 7, broadly subcarinate dorsal subapical sinuation; apical elytral seta present, subapical seta absent; lateral elytral setae (5–6) + (4–5); elytral marginal depression narrow with little upraised margin at humerus, slightly broader laterally, margin beaded anterad subapical sinuation. *Mesepisternum* densely punctate anteriorly, ~14 large punctures in 2–3 rows; metepisternum slightly longer than broad, width to length ratio 0.75; metepisternum separated from metepimeron by distinct suture. *Abdomen* with visible ventrites 1–5 irregularly wrinkled laterally, ventrites 3–6 with rounded depressions laterally; suture between visible ventrites 2 and 3 effaced laterally. *Metatarsomere 4* indistinctly lobate, overall length including lobes 1.5× median tarsomere length, both subapical and apical setae present; metatarsal dorsolateral sulci very shallow, lateral, median surface of tarsomere broadly convex. *Microsculpture* obsolete on frons, surface glossy, shallow isodiametric sculpticells in transverse rows on neck; pronotal disc glossy, micro- sculpture obsolete but indistinct transverse sculpticells, 3–4× broad as long, discernible just outside areas of light reflection; pronotal median base with indistinct isodiametric mesh between punctures; elytral disc glossy, obsolete transverse mesh visible along edge of reflected light; elytral apex with shallow isodiametric and transverse sculpticells; metasternum with distinct transverse mesh; laterobasal abdominal ventrites covered with swirling isodiametric and transverse sculpticells. *Coloration* of head capsule rufous with slight piceous cast; antennomeres 1–3 rufoflavous, 4–11 slightly darker, the apical antennomeres rufobrunneous; pronotal disc dark rufous, pronotal margins with piceous cast, basal and apical edge slightly paler, rufobrunneous where narrowed in thickness; proepipleuron rufoflavous medially, dark dorsally, rufous with piceous cast ventrally to match proepisternum; elytral disc dark rufous, sutural interval rufous basally, rufoflavous apically; elytral marginal depression concolorous at humerus, rufoflavous in deepest portion laterally and to subapical sinuation; elytral apex broadly slightly paler, rufobrunneous; elytral epipleuron rufoflavous ventrad dark margin, rufobrunneous ventrally, metepisternum slightly darker, rufous with piceous cast; abdomen dark rufous basally, ventrites 4–6 paler, rufobrunneous, apical ventrite with apical third paler, rufoflavous; metafemur rufoflavous; metatibia rufoflavous with brunneous cast.

**Male genitalia.** (n = 1). Aedeagal median lobe evenly curved and of subequal diameter in basal half, narrowed apically to tightly rounded apex that extends little beyond apical ostial margin (Fig. 4E); internal sac lightly sclerotized, only flagellar plate visible in uneverted dissection, length of plate 0.33× distance from parameral articulation to apex; right paramere short, apex extended toward apex 0.7× distance from parameral articulation to apex, left paramere longer, extended 0.9× that distance.

**Female reproductive tract.** (n = 1). Bursa copulatrix very short, present as a very short lobe situated dorsad the broad common oviduct (Fig. 5C); bursal apex extended beyond evident transverse fold at base of oviduct the same distance as that from transverse fold to a line drawn between bases of basal gonocoxites; bursal surface lightly sclerotized, as membranous as surface of median oviduct; spermatheca reniform, spermathecal gland connected to base of spermatheca by a short duct; basal gonocoxite 1 with apical fringe of 3–4 setae, and 10–11 smaller setae along mesal margin, spanning ventromedial to dorsomedial surfaces of gonocoxite (Fig. 6C); apical gonocoxite 2 narrow basally with tightly rounded apex, single dorsal and lateral ensiform setae and 2 apical nematiform setae.

**Holotype** male (MNHN) labeled: French Polynesia: Moorea / Tohiea summit el. 1080- / 1120 m 12-IX-2006 lot 09 / 17°33.07'S, 149°49.38'W / beating dead fern fronds / C.P. Ewing // HOLOTYPE / Mecyclothorax / popotioaoa / J.K. Liebherr 2012 (black-bordered red label).

**Allotype** female (MNHN): French Polynesia: Moorea / Tohiea summit el. 1120 m / 12-IX-2006 lot 08 / 17°33.07'S, 149°49.38'W / beating ferns C.P. Ewing // ALLOTYPE / Mecyclothorax / popotioaoa / J.K. Liebherr 2012 (black-bordered red label).

##### Other paratypes.

SOCIETY ISLANDS. Moorea: Tohiea summit, 1120 m el., S17°33.07', W149°49.38', 12-ix-2006 lot08, beating ferns, Ewing (CUIC, 1); 1120 m el., S17°33.07', W149°49.38' 12-ix-2006 lot 10, pyrethrin fog mossy log, deep gulch, Polhemus (NMNH, 1); gulch S of summit, 1160-1180 m el., S17°33.03', W149°49.36', 24-ix-2009, on fern frond, gulch wall, MBIO 5852, Ewing & Yang (EMEC, 1).

##### Etymology.

The species epithet is a compounding of the Tahitian word popoti, beetle or cockroach, and oaoa, the Tahitian adjective narrow (Wahlroos, 2002), signifying the constricted pronotal base and narrow body of adult beetles of this species. As oaoa is indeclinable, the epithet is to be treated as a noun in apposition.

**Distribution and habitat.** Four of the five specimens recorded for the species have been collected on fern fronds, either living or dead, in all such instances associated with individuals of *Mecyclothorax mapo*. The fifth specimen was collected in association with *Mecyclothorax mapo* and the single known *Mecyclothorax menemene* by pyrethrin fogging of a mossy log complex in a deep, wet gulch.

### *Mecyclothorax viridis* species group

**Diagnosis.** Perrault (1986) assigned four species to this group, all of which exhibit: 1, cordate pronotum with sinuate basolateral margins, and right, setose hind angles; 2, distinctly convex elytra with upraised humeral margin; and 3, presence of 1 or 2 dorsal elytral setae. He noted that the four species comprise two geographic pairs, *Mecyclothorax balli* Perrault and *Mecyclothorax castaneus* Perrault from Marau, and *Mecyclothorax ata* Perrault and *Mecyclothorax viridis* Perrault from adjacent Aorai. Subsequently, four undescribed species have been found on Pito Hiti, the triangular summit ENE of Mont Orohena (E.M. Claridge, pers. comm.). Another undescribed species possessing the above characteristics, and thus assignable to this group upon description, has been collected on Mauru, a peak isolated far to the east of Orohena by the broad valley of the Papenoo River and tributaries (Liebherr in press).

#### Mecyclothorax mapo sp. n.

urn:lsid:zoobank.org:act:6E037A49-8874-4791-BCC4-F3FD8EC5556F

http://species-id.net/wiki/Mecyclothorax_mapo

##### Diagnosis.

This species shares transverse-line elytral microsculpture and deep punctate elytral striae with *Mecyclothorax castaneus*, and individuals are of similar body size; standardized body length for this species 3.8–4.4 mm versus 3.8 mm for *Mecyclothorax castaneus*. The pronotum is of similar dimensions in the two species, with MPW/PL = 1.14–1.17 (n = 5) in this species, versus a ratio of 1.19 in *Mecyclothorax castaneus* (Perrault, 1986). The species differ in setation, with this species consistently characterized by two discal elytral setae, and therefore a setal formula of 2221, versus *Mecyclothorax castaneus* where one of the two type specimens had two discal setae on one elytron, whereas the other three elytra of the two beetles were unisetose. In addition, the pronotal base of this species is relatively broader, MPW/BPW = 1.52–1.64 (n = 5), versus a narrower base and greater ratio of 1.70 for *Mecyclothorax castaneus*.

##### Description.

*Head capsule* withfrontal grooves nearly straight on lateral margins, depressed area of frons triangular with broadest portion at frontoclypeal suture, groove terminated posteriorly at thin carina mesad anterior supraorbital seta; dorsum of head flat on frons in lateral view, neck convex; ocular lobe broadly convex, little protruded from head capsule, hind portion meeting gena at 135˚ angle, at a fine groove bordered posterad by fine carina; compound eye with 15–16 ommatidia along a horizontal diameter defined by lower margin of antennal articulatory socket; ocular ratio 1.40–1.49 (n = 5), ocular lobe ratio 0.73–0.83 (n = 5); labral anterior margin nearly straight, only slightly emarginate; antennomeres 1–3 glabrous except for apical setae; antennae submoniliform, antennomere 8 length 1.6× greatest breadth; mentum tooth sides defining an acute angle, apex subacuminate. *Pronotum* quadrisetose, cordate (Fig. 3C), margin variously, slightly convergent, subparallel, or slightly divergent anterad obtuse hind angles; median base depressed relative to disc, margined anteriorly by a row of punctures, some elongate, 13–14 punctures each side of base, punctures sparser medially; basal margin convex between the laterobasal depressions; median longitudinal impression fine, shallow but continuous on disc, continued as fine impression onto front of median base; anterior transverse impression broad, shallow, smooth, finely incised only mesad front angle; anterior callosity only slightly convex, with very fine and shallow longitudinal wrinkles, variously restricted to front margin of pronotum to crossing callosity and anterior transverse impression; front angles slightly protruded, tightly rounded, apical and basal pronotal widths subequal, APW/BPW = 1.0–1.09 (n = 5); lateral marginal depression very narrow, edge beaded throughout most of length, except at front angle where margin is slightly upturned to flat, and along basolateral sinuation where margin is broadly upturned; laterobasal depression defined by linear mesal extension of lateral depression and lateral raised basolateral margin, punctures of median base not reaching deepest linear portion; proepisternum with 6 distinct punctulae along hind margin, ~5 smaller punctures along marginal collar of proepimeron; prosternal process narrowly depressed between broadly upraised lateral areas between procoxae, convex posterad at juncture with posterior face. *Elytra* subovoid, sides broadly convex; disc convex with sides sloping to near vertical juncture with lateral marginal depression; basal groove short, distinctly, anteriorly curved to proximate, angulate humeri, MEW/HuW = 2.29–2.43 (n = 5); parascutellar seta present; parascutellar striole 3–4 punctate, irregularly depressed or not between punctures; sutural interval dorsally expanded to meet at a sutural callous that extends to apex; striae 1–4 distinctly impressed on disc, elongately punctate at depth, striae 5–6 shallower but also elongately punctate, stria 7 reduced to series of isolated punctures; sutural stria 1 deep and smooth apically, stria 7 nearly as deep apicad subapical sinuation, 2–6 shallow, continuous, with rudimentary punctures in deeper portions; intervals 2–6 slightly convex on disc; interval 8 bulbously carinate laterad stria 7 dorsad subapical sinuation, the interval nearly vertical in orientation laterad its midpoint; two dorsal elytral setae in impressions that span ½ to ⅔ of interval 3, positioned at 0.32–0.34× and 0.66–0.68× elytral length; apical elytral seta present, subapical seta absent; lateral elytral setae 6 + 5; elytral marginal depression narrow, edge thick and upturned at humerus, more thinly upturned laterally, beadlike anterad subapical sinuation; subapical sinuation shallowly excavate, broad. *Mesepisternum* anteriorly with 6 large, isolated punctures in 1–2 rows; metepisternum longer than wide, width to length ratio 0.72; metepisternum separated from metepimeron by distinct suture; metathoracic wing vestigium an elongate strap, 1.57× long as wide that extends from base to 0.73× length of metanotum, rudiments of R and M veins visible on strap. *Abdomen* with irregular wrinkles laterally on visible ventrites 1–4, rounded depressions laterally on ventrites 3–6; suture between visible ventrites 2 and 3 laterally effaced. *Metatarsomere 4* triangular in dorsal view, lobate, length including lobes 1.6× median tarsomere length, subapical and apical setae present; metarsal dorsolateral sulci shallow, lateral, tarsomeres broadly convex medially. *Microsculpture* of frons reduced, surface glossy, shallow transverse mesh visible adjacent to area of reflected light; pronotal disc glossy, shallow, indistinct transverse mesh, sculpticells 3–4× broad as long, visible adjacent to area of reflected light; pronotal median base glossy between punctures; elytral disc with distinct, subiridescent transverse mesh, sculpticells 3–4× broad as long, mixed with less cross–connected transverse lines; elytral apex with transverse mesh, sculpticells 2–3× broad as long; metasternum glossy with obsolete transverse mesh; laterobasal abdominal ventrites glossy, covered with shallow swirling isodiametric and transverse mesh. *Coloration* of head capsule rufobrunneous with piceous cast; antennomeres 1–2 flavous, 3–4 rufoflavous, 5–11 slightly darker; pronotal disc rufobrunneous, anterior transverse impression rufopiceous; proepipleuron rufoflavous, proepisternum rufobrunneous; elytral disc rufobrunneous with silvery to bluish reflection; sutural interval rufous basally, rufoflavous apically; elytral lateral marginal depression concolorous with disc at humerus, increasingly paler to rufoflavous anterad subapical sinuation; elytral apex narrowly rufoflavous anterad incised portion of stria 7; elytral epipleuron rufoflavous, metepisternum rufobrunneous; abdomen rufobrunneous, lateral margins concolorous to darker, with piceous cast, abdominal apical ventrite rufoflavous in apical ¼; metafemur flavous with medial brunneous cast; metatibia flavous.

**Male genitalia.** (n = 3). Aedeagal median lobe broad in basal ⅔ of length, narrowed apically to broadly rounded apex with blunt apical face, ventral portion of median lobe straight (Fig. 4F); internal sac ventrally covered with dense microspiculate field, distinct ventral or dorsal microtrichial patches absent; flagellar plate large, length 0.57× distance between parameral articulation and apex, gonopore visible on middle of dorsal surface, longitudinally radiate sclerotic ridges on inner, ventral, surface of plate; right paramere narrowly elongate (Fig. 4G), tip extended 0.85× distance from parameral articulation to apex, left paramere slightly longer, extended 0.90× such distance.

**Female reproductive tract.** (n = 1). Bursa copulatrix constricted basally apicad juncture with common oviduct (Fig. 5D), bursa columnar, length 3× greatest breadth in slide–mounted dissection, surface thin, membranous based on staining with Chlorazol Black; spermatheca reniform, spermathecal duct heavily sclerotized, inflexible; spermathecal gland attached to spermatheca by an elongate duct; basal gonocoxite 1 with 2–3 apical fringe setae, and 2–3 smaller setae along the mesal surfaces of coxite (Fig. 6D); gonocoxite 2 broad basally, subacuminate apically, the lateral margin broadly concave; 2 lateral ensiform setae, 1 dorsal ensiform seta, and 2 apical nematiform setae present on gonocoxite 2.

**Holotype** male (MNHN), labeled: French Polynesia: Moorea / Tohiea summit el. 1125– / 1200 m 12–IX–2006 lot 01 / 17°33.03'S, 149°49.33'W / beating ferns & *Myrsine* / J.K. Liebherr // HOLOTYPE / Mecyclothorax / mapo / J.K. Liebherr 2012 (black–bordered red label).

**Allotype** female (MNHN), labeled as holotype but with black–bordered red ALLOTYPE label.

##### Other Paratypes.

SOCIETY ISLANDS. Moorea: Tohiea summit, 1125–1200 m el., S17°33.03', W149°49.33', 12–ix–2006 lot 01, beating *Myrsine* + ferns, Liebherr (CUIC, 8); 1125 m el., S17°33.07', W149°49.38', 12–ix–2006 lot 02, pyrethrin fog *Weinmannia* moss + roots, Liebherr (CUIC, 3); 1120 m el., S17°33.07', W149°49.38', 12–ix–2006 lot 03, beating *Dicranopteris* ferns, Liebherr (CUIC, 5); lot 05, beating rotten *Freycinetia*, Liebherr (CUIC, 4); 1150–1200 m el., S17°33.03', W149°49.33', 12–ix–2006 lot 07, beating flowering *Myrsine* at night, Liebherr (CUIC, 12; NMNH, 2); 1120 m el., S17°33.07', W149°49.38', 12–ix–2006 lot 08, beating ferns, Ewing (EMEC, 2); 1120 m el., S17°33.07', W149°49.38', 12–ix–2006 lot 09, dead fern fronds, deep gulch, Ewing (CUIC, 2; EMEC, 2); 1120 m el., S17°33.07', W149°49.38', 12–ix–2006 lot 10, pyrethrin fog mossy log, deep gulch, Polhemus (NMNH, 4); gulch S of summit 1160–1180 m el., S17°33.03', W149°49.36', 24–ix–2009 on fern frond, gulch wall, MBIO 5852, Ewing & Yang (EMEC, 5); muddy gulch on trail, 1150 m el., S17°33.08', W149°49.31', 24–ix–2009, pyrethrin fog mossy tree trunk, MBIO 5856, Ewing (CUIC, 1; EMEC, 1); summit along ridge to west, 1190–1207 m el., S17°33.04', W149°49.34', 24–ix–2009, beating *Myrsine*, MBIO 5857, Ewing (CUIC, 1; EMEC, 6); muddy gulch on trail, 1150 m el., S17°33.08', W149°49.31', 24–ix–2009, beating *Angiopteris evecta*, MBIO 5854, Stavrinides (EMEC, 2); 1170 m el., S17°33.08', W149°49.31', 25–ix–2009, pyrethrin fog mossy tree, MBIO 5853, Ewing (EMEC, 2); 1150 m el., S17°33.08', W149°49.31', 25–ix–2009, pyrethrin fog mossy tree trunk, MBIO 5859, Ewing (EMEC, 1); gulch S of summit, 1150–1170 m el., S17°33.03', W149°49.36', 26–ix–2009, on fern frond steep gulch, MBIO 5861, Ewing (EMEC, 3).

##### Etymology.

Given that this species is most similar to *Mecyclothorax castaneus*, the common name of the Tahitian chestnut tree, *Inocarpus fagifer* (Parkinson) (Fabaceae)—i.e. mapo (Wahlroos, 2002)—was chosen for the species epithet. The epithet is to be treated as a noun in apposition.

##### Distribution and habitat.

This species has been found in a variety of situations on Mont Tohiea, accounting for 68 of the 90 specimens of *Mecyclothorax* collected on or near the summit. Specimens have been found by sampling ferns, *Angiopteris*, rotten *Freycinetia*, *Myrsine* foliage and flowers, and moss–covered *Wienmannia* trunks and roots. In keeping with this species’ numerical dominance, it has been collected in association with all other *Mecyclothorax* spp. known from Mont Tohiea.

#### Mecyclothorax fatata sp. n.

urn:lsid:zoobank.org:act:14EE6095-7917-4D0F-8D25-42F7C8763199

http://species-id.net/wiki/Mecyclothorax_fatata

##### Diagnosis.

This species shares upturned pronotal margins with a visible lateral depression (Fig. 3D) and regular transverse–mesh elytral microsculpture with *Mecyclothorax ata* Perrault. Individuals of the two species are of similar body size; standardized body length 4.7–5.0 for this species versus 4.5 mm for *Mecyclothorax ata*. However this species deviates from *Mecyclothorax ata* by presence of only the anterior elytral seta resulting in a setal formula of 2211 versus 2221 for *Mecyclothorax ata*. The pronotal base is also more constricted in this species, MPW/BPW = 1.53–1.62 (n = 5) versus a ratio of 1.49 in *Mecyclothorax ata* (Perrault 1986).

##### Description.

*Head capsule* gracile, elongate, frontal grooves subparallel at thin carina posteriorly, mesad anterior supraorbital seta, convergent anterad, clypeo–ocular prolongation broadly convex, frons between groove densely covered with fine transverse wrinkles; dorsum of head flat on frons in lateral view, neck convex; ocular lobe protruded, posterior portion meeting gena at broad, moderately deep groove; compound eye slightly protruded from ocular lobe, slightly convex dorsally laterad supraorbital seta, more than 20 ommatidia along diameter defined by lower margin of antennal articulatory socket; ocular ratio 1.48–1.60, ocular lobe ratio 0.79–0.86; labral anterior margin broadly shallowly emarginate ¹�₉ length; antennomeres 1–3 mostly glabrous except for apical seta, antennomere 3 with a few very short setae on posterior surface of shaft; antennae moderately elongate, antennomere 8 length 1.8× maximum breadth; mentum tooth sides defining an acute angle, apex tightly rounded. *Pronotum* smoothly cordate, basolateral margins nearly subparallel anterad rounded obtuse hind angles, distinctly divergent just anterad basal pronotal setae; pronotum somewhat transverse, MPW/PL = 1.15–1.20 (n = 5); median base distinctly depressed relative to disc, punctures arrayed along margin of base and disc, 13–14 punctures of various sizes each side; basal margin slightly convex between laterobasal depressions; median longitudinal impression very fine and shallow but complete on disc, prolonged as longitudinal crease on median base; anterior transverse impression broad and shallow, smooth, finely incised only mesad front angles; anterior callosity flat, little upraised, densely covered with shallow longitudinal wrinkles; front angles very slightly protruded anterad, broadly rounded, distances between front and hind angles subequal, APW/BPW = 0.99–1.05 (n = 5); lateral marginal depression bordered by upraised lateral margin at lateral pronotal seta, broader with margin less upraised at front angle, broader basally joining laterobasal depression which is broader, the surface irregularly punctured; proepisternum with 7 distinct punctulae along hind margin, proepimeron with about 10 very small punctures along marginal collar; prosternal process narrowly depressed medially, sides broadly upraised between procoxae, surface convex posterad at juncture with posterior face. *Elytra* ellipsoid, humeri proximate, disc convex medially, surface sloping laterally to near vertical juncture with lateral marginal depression; basal groove briefly curved to angulate humerus, MEW/HuW = 2.43–2.67 (n = 5); parascutellar seta present; parascutellar striole 3–4 punctate, surface not depressed between punctures; sutural intervals elevated to meet at suture, more convex, callouslike apically; striae 1–6 punctate, the punctures expanding strial width, but striae shallow between punctures, and striae progressively shallower laterally, stria 7 obsolete, indicated by irregularly evident shallow, longitudinal punctulae; intervals on disc flat; sutural stria 1 and stria 7 deep, well defined at apex, striae 2–6 very shallow, difficult to trace; mesal margin of interval 8 protruded as a distinct carina apicad the subapical sinuation; anterior dorsal elytral setae in small impression spanning ½ of interval 3, the setae situated at 0.25–0.26× elytral length; apical elytral seta present, subapical seta absent; lateral elytral setae 7 + 6; elytral marginal depression moderately narrow, edge upturned at humerus, depression broader and edge more upturned laterally to elytral midlength, then depression narrowed, margin beadlike anterad subapical sinuation; subapical sinuation very shallow and brief, nearly obsolete. *Mesepisternum* with single dorsoventral row of 5–6 large punctures; metepisternum moderately elongate, width to length ratio 0.68; metepisternum separated from metepimeron by distinct suture; metathoracic flight wing vestigium an elongate strap, length 3× width, the apical half of strap extended beyond hind margin of metanotum, with rudiments of wing veins C, R, M, and Cu visible in vestigium. *Abdomen* with visible ventrites 1–4 irregularly wrinkled laterally, ventrites 3–6 with rounded depressions laterally; suture between visible ventrites 2 and 3 effaced laterally. *Metatarsomere 4* emarginate apically, short apical lobes present, overall tarsomere length 1.5× median tarsomere length; metatarsomere 4 with long apical and very short subapical setae that are situated along dorsal margin of tarsal apical lobes; metatarsal dorsolateral sulci shallow and lateral, tarsomere dorsum broad, nearly flat. *Microsculpture* on frons a regular transverse mesh, sculpticell breadth 2–3× length, neck with isodiametric sculpticells in transverse rows; pronotal disc with a mixture of transverse lines and transverse mesh with sculpticell breadth 3–4× length, the surface subiridescent due to microsculpture; pronotal median base covered with dense, swirling isodiametric and transverse sculpticells between punctures; elytral disc with distinct transverse mesh, sculpticell breadth 2–4× length, the surface subiridescent; elytral apex with transverse mesh, sculpticells 2–4× broad as long, the sculpticells slightly upraised; metasternum with distinct elongate transverse mesh; laterobasal abdominal ventrites with glossy surface, swirling isodiametric and transverse sculpticells plainly visible. *Coloration* of head capsule rufobrunneous with a piceous cast; antennomeres 1–2 flavous, 3–11 rufoflavous; pronotal disc rufobrunneous with silvery metallic reflection, lateral margins, base and anterior callosity darker than disc, with piceous cast; proepipleuron rufoflavous, proepisternum rufobrunneous; elytral disc rufobrunneous with silvery reflection; sutural interval concolorous with disc basally paler, rufoflavous apically; elytral marginal depression concolorous with disc at humerus, depressed area rufoflavous from midlength to subapical sinuation; elytral epipleuron rufoflavous, metepisternum rufobrunneous; abdomen rufobrunneous, broadly paler apically to rufoflavous apex of visible ventrite 6; metafemur rufoflavous; metatibia rufoflavous with brunneous cast.

**Male genitalia.** (n = 2). Aedeagal median lobe of equal diameter in basal ⅔ of length, narrowed to a bluntly rounded, ventrally expanded apex (Fig. 4H); internal sac with melanized microspicules on surface, appearing dark in uneverted dissection; flagellar plate large, melanized, length 0.5× distance from parameral articulation to apex; right paramere narrowly elongate, apex extended 0.85× distance from parameral articulation to apex, left paramere longer, apex extended 0.90× such distance.

**Female reproductive tract.** (n = 1). Bursa copulatrix columnar, length 2.4× greatest width in microslide–mounted dissection (Fig. 5E), bursal surface lightly sclerotized based on staining with Chlorazol Black; spermatheca reniform, spermathecal duct thin and lighly sclerotized; spermathecal gland bulbous, apparently filled with material that did not clear in 10% KOH in dissected individual, attached to spermatheca by short duct; basal gonocoxite 1 with apical fringe of 4 setae (Fig. 6E), 13–14 small setae arrayed across medial portion of coxite; apical gonocoxite 2 broad basolaterally, the lateral margin broadly excavate, the apex subacuminate; 2 lateral and 1 dorsal ensiform setae, and 2 apical nematiform setae present.

**Variation.** Marginal setation of the apical visible ventrite in males is unstable in this species. Of the four male specimens, one individual has 4 terminal abdominal setae along the apical margin of visible ventrite 6, 2 on each side (EMEC); one has 3 apical setae, 1 on the right and 2 on the left (CUIC); and two others exhibit the usual 2 setae, 1 each side (MNHN, EMEC).

**Holotype** male (MNHN), labeled: FRENCH POLYNESIA: / Moorea Tohiea summit / 12–IX–2006 lot 07 / S17°33.03', W149°49.33' / el. 1150–1200 m beating / flowering *Myrsine* after / dark J.K. Liebherr // HOLOTYPE / Mecyclothorax / fatata / J.K. Liebherr 2012 (black–bordered red label).

**Allotype** female (MNHN), labeled as holotype but with black–bordered red ALLOTYPE label.

##### Other Paratypes.

SOCIETY ISLANDS. Moorea: Tohiea summit, 1125–1200 m el., S17°33.03', W149°49.33', 12–ix–2006 lot 01, beating *Myrsine* + ferns, Liebherr (CUIC, 2); 1125 m el., S17°33.07', W149°49.38', 12–ix–2006 lot 02, pyrethrin fog *Weinmannia*, moss + roots, Liebherr (CUIC, 1); summit along ridge to west, 1190–1207 m el., S17°33.04', W149°49.34', 24–ix–2009, beating *Myrsine*, MBIO 5857, Ewing (EMEC, 1); muddy gulch on trail, 1170 m el., S17°33.08', W149°49.31', 25–ix–2009, pyrethrin fog mossy tree, MBIO 5853, Ewing (CUIC, 1); 1150 m el., S17°33.08', W149°49.31', 25–ix–2009, pyrethrin fog mossy tree trunk, MBIO 5859, Ewing (EMEC, 1).

##### Etymology.

Because this species is most similar to *Mecyclothorax ata*, the Tahitian epithet fatata, near or nearly (Wahlroos 2002) was chosen to express the similarity. The epithet is indeclinable and is to be treated as a noun in apposition.

##### Distribution and habitat.

All six collections and eight specimens of *Mecyclothorax fatata* were made in association with the numerically dominant *Mecyclothorax mapo*. In two instances *Mecyclothorax perraulti* was also present in the sample. Five of the eight *Mecyclothorax fatata* specimens were collected from *Myrsine*, one from moss–covered *Weinmannia*, and two others from unidentified trees.

## Discussion

The greatest similarity of the seven Moorean *Mecyclothorax* spp. to seven different Tahitian *Mecyclothorax* spp. (Table 1) points to independent biogeographic relationships—i.e. independent speciation events—for the seven sister–species pairs. This pattern suggests that all seven speciation events have occurred such that the descendant species occupy allopatric distributions on either side of the Moorea Channel. Isolation by this Channel leading to speciation could have conceivably involved three biogeographic phenomena: 1, vicariance between Moorea and Tahiti based on subsidence of intermediate land areas and associated oceanic incursion; 2, dispersal of an ancestral population from Tahiti to Moorea; 3, dispersal of an ancestral population from Moorea to Tahiti. The first option involving subsidence of an ancient mountain range was favored by Crampton (1917, 16; 1932, 194) in his interpretation of the biogeographic history of *Partula* land snails. Minimum oceanic depths between Moorea and Tahiti are more than 1500 m (U.K. Hydrographic Office 2012). Currently there are no data on the amount of subsidence by Moorea caused by Pacific Plate loading associated with the shield–building phase of the younger volcanoes of Tahiti. However, data from the Hawaiian Islands may be pertinent. During the development of Hawaii Island, subsidence levels of 1300–2000 m occurred along the Hana Ridge to the east of Maui Island (Price and Elliott–Fisk 2004; Faichney et al. 2010), the next youngest island in the Hawaiian chain. If the Tahiti–Moorea island pair interacted similarly at their position on the Pacific Plate, Crampton’s scenario may be correct. Submarine mapping of ancient reef systems surrounding Moorea and Tahiti is required to further evaluate the geological underpinnings for this option.

**Table 1.** Moorean *Mecyclothorax* spp. and hypothesized Tahitian adelphotaxa based on greatest morphological similarity, plus distributional range in Tahiti of hypothesized adelphotaxa. The distributional ranges Aorai and Marau are the ridges culminating in those peaks to the south. Taiarapu represents species collected in the Mts. Teatara, Presqu’île de Tairarapu.<br/>

**Moorean species**	**Tahitian adelphotaxon**	**Adelphotaxon range**
*Mecyclothorax perraulti*	*Mecyclothorax gourvesi*	Aorai + Marau
*Mecyclothorax pahere*	*Mecyclothorax paraltiusculus*	Taiarapu
*Mecyclothorax menemene*	*Mecyclothorax jarrigei*	Aorai
*Mecyclothorax mahatahi*	*Mecyclothorax cupripennis*	Taiarapu
*Mecyclothorax popotioaoa*	*Mecyclothorax hemisphaericus*	Marau
*Mecyclothorax mapo*	*Mecyclothorax castaneus*	Marau
*Mecyclothorax fatata*	*Mecyclothorax ata*	Aorai

Taking up the hypotheses that the biogeographic relationships of the Moorean and Tahitian taxa are due to dispersal, we can ask whether a consistent biogeographic relationhip between Mont Tohiea and an area of endemism in Tahiti is observed. If *Mecyclothorax* beetles dispersed between these islands, they necessarily did so over water, not in the air, as all *Mecyclothorax* spp. on both islands possess vestigial flight wings and a truncated metathoracic flight apparatus evolutionarily associated with winglessness (Darlington 1936). Given that restriction, dispersal would most likely have been associated with flooding events, landslides, and subsequent rafting between islands on vegetative masses broken loose from the very steep, highly eroded volcanic flanks present on both islands (Zimmerman 1948). Marau and Aorai represent the Tahitian mountain ridges most proximate to Moorea, and thus most likely to be implicated in dispersal to or from Moorea. Based on the distributional ranges of the Tahitian adelphotaxa (Table 1), there is majority support for this pattern as five of the seven species occur on either Aorai or Marau. However two of the adelphotaxa are restricted to Taiarapu, a geographically distant source or recipient.

The generality of the proposed, most prevalent dispersal pattern can be tested using information from other taxa. To date the best example of such a test involves the Moorean Nabidae, which comprise three micropterous species, all related to different micropterous species in Tahiti (Polhemus 2010). *Nabis tohiea* Polhemus is the putative adelphotaxon to *Nabis orohena* Polhemus, the latter broadly distributed on Marau, Aorai, Orohena, Pito Hiti, Taiarapu, and Mont Mauru on the eastern versant of Tahiti Nui. *Nabis mooreana* Polhemus is considered closest to *Nabis tangaroa* Polhemus, with the Tahitian *Nabis tangaroa* known only from Marau. And the third Moorean species, *Nabis polynesica* Polhemus, is sister to *Nabis tahitiensis* Polhemus, a species recorded from Marau, Mauru, and Taiarapu. Based on information from *Nabis* spp., Marau is represented in the distributions of all three Tahitian adelphotaxa of the Moorean taxa, consistent with a dispersal scenario involving over–water transport on vegetative debris cut loose by floodwater induced land slips.

At present we have no evidence that Moorean *Mecyclothorax* have undergone autochthonous speciation in Moorea. Hoch (2006) proposed that two Moorean species of *Oteana* Hoch (Hemiptera: Cixiidae) are adelphotaxa, suggesting that the island has had a complicated enough geographical and botanical history during its 1.52 Myr subaerial lifespan (Guillou et al. 2000) to have supported speciation within its bounds. Recent collections of *Mecyclothorax* beetles have all occurred near the summit of Mont Tohiea, from 1100–1207 m elevation. During his Tahitian collecting, Perrault (1986, 1987, 1988) found singleton specimens he described as three species—*Mecyclothorax teatara* Perrault, *Mecyclothorax ferruginosus* Perrault, and *Mecyclothorax sinuatus* Perrault—in habitats from 800–1000 m. During the 2006 survey that resulted in the first collections of *Mecyclothorax* on Mont Toheia, another undescribed *Mecyclothorax* was discovered in a riparian habitat at 705 m elevation on Mont Mauru in eastern Tahiti Nui (unpubl. data). Thus 700 m elevation stands as a likely lower limit for present–day distributions of *Mecyclothorax* beetles on Tahiti. Assuming a similar lower elevational limit for taxa in Moorea suggests that Moorea’s Mt. Rotui, isolated by low-lying valleys and peaking at 899 m elevation, is the locality most likely to contain Moorean adelphotaxa to one or more of the seven species precinctive to Mont Tohiea. Other peaks on the main Mont Toheia massif—e.g. Mont Mouaroa—may also support *Mecyclothorax*, though this summit at 880 m elevation is connected to Mont Toheia by a ridgeline supporting montane forest, and thus would be less likely to house distinct species.

**Figure 7.**
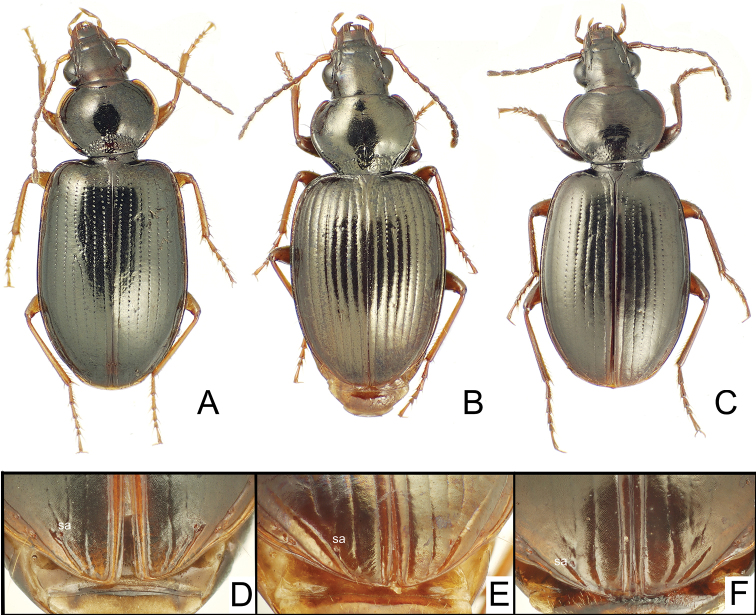
**A–C** *Mecyclothorax* spp., females, dorsal view. **A** *Mecyclothorax punctipennis*, Mt. Buffalo S.P., Victoria, Australia; proposed adelphotaxon to both the Society Island and Hawaiian *Mecyclothorax* radiations **B** *Mecyclothorax wallisi*, female, Aorai, Tahiti; member of *Mecyclothorax striatopunctatus* species group **C** *Mecyclothorax montivagus*, Haleakala, Maui; proposed as closest extant relative to founding Hawaiian *Mecyclothorax* species **D–F** Elytral apex, dorsal view; sa, subapical elytral seta **D** *Mecyclothorax punctipennis* **E** *Mecyclothorax wallisi* **F** *Mecyclothorax montivagus*; subapical seta absent from right elytron.

**Figure 8.**
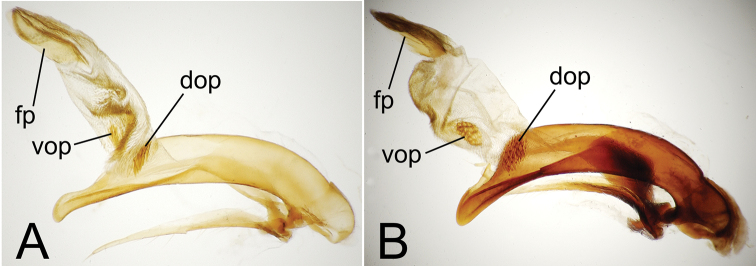
*Mecyclothorax* spp., male aedeagal median lobe and parameres, internal sac everted. **A** *Mecyclothorax punctipennis*. **B** *Mecyclothorax montivagus*. Abbreviations: **dop** dorsal ostial microtrichial patch; **fp** flagellar plate; **vop** ventral ostial microtrichial patch.

The most speciose radiations of *Mecyclothorax* spp. are concentrated in the Society and Hawaiian island chains. Is this shared level of extreme diversity indicative of phylogenetic affinity for these two radiations? The most generalized species in the genus are Australian. Moore (1984) redescribed the two most widespread Australian *Mecyclothorax*—*Mecyclothorax punctipennis* (Macleay) and *Mecyclothorax ambiguus* (Erichson)—and clarified their nomenclatural status. Both species inhabit broad geographic ranges in Australia. *Mecyclothorax punctipennis* occurs in dry to mesic, subtropical lowland to montane forest and is found in both West Australia and along the east coast, whereas *Mecyclothorax ambiguus* is restricted to wetter more temperate forests from New South Wales to Tasmania. Both species comprise flight–capable individuals living in a wide variety of habitats. Of these two, Britton (1948) proposed the Australian *Mecyclothorax punctipennis* (his “*Mecyclothorax ambiguus*” prior to Moore’s [1984] clarification) (Fig. 7A) as the species most similar to the generalized Hawaiian *Mecyclothorax montivagus* (Blackburn) (Fig. 7C) based on pronotal, elytral and aedeagal configurations (Fig. 8). He considered *Mecyclothorax montivagus* to represent the most generalized Hawaiian *Mecyclothorax*. Recent study confirms his conclusion. Among the entirely brachypterous Hawaiian *Mecyclothorax* fauna, *Mecyclothorax montivagus* exhibits the largest flight–wing vestigium, with the wing rudiment a narrowed stenopterous strap of length 3.3× breadth, and including rudiments of the costa, radius, medius, and cubitus wing veins. This strap extends about 0.40× its length past the posterior margin of the metanotum. No other Hawaiian *Mecyclothorax* species has a flight wing rudiment this well developed (unpubl. data). Thus best evidence points to *Mecyclothorax montivagus* as the most generalized extant Hawaiian species, and it is hypothesized to be the closest phylogenetic descendant of the ancestral Hawaiian *Mecyclothorax* propagule. Choosing among potential extant *Mecyclothorax* spp. from the southwest Pacific to have colonized Hawaii, *Mecyclothorax punctipennis* of Australia represents the morphologically most similar and ecologically most appropriate candidate.

Based on comparison of the seven new Moorean species plus all Tahitian species described by G.G. Perrault (MNHN) to representatives of all Australian and New Zealand species (Moore et al. 1987; Baehr 2009; Liebherr and Marris 2009), the greatest morphological similarity also links Australia’s *Mecyclothorax punctipennis* to the Tahitian members of the *Mecyclothorax striatopunctatus* species group (Perrault 1986) (Fig. 7B). The four *Mecyclothorax striatopunctatus* group species all display the maximal setal formula of 2222, as does *Mecyclothorax punctipennis*. Members of the *Mecyclothorax striatopunctatus* species group also exhibit an ovoid pronotum with little projected hind angles, and punctate elytral striae. Being vestigially winged, the elytra have more rounded humeri, as also observed in *Mecyclothorax montivagus* (Figs 7B, C), and the elytral striae are the deepest of the three, with lateral striae more well developed than in *Mecyclothorax punctipennis* or *Mecyclothorax montivagus* (Fig. 7). Aedeagal conformation is also very similar in males of all three species. The aedeagal lobe of the Tahitian species terminates in moderately expanded apex (Perrault 1986, figs 22, 23) similar to the aedeagal median lobe of *Mecyclothorax punctipennis* (Fig. 8A) and *Mecyclothorax montivagus* (Fig. 8B). The internal sac of the latter two taxa are also similar (data for the Tahitian species not available), sharing: 1, robust, moderately elongate shape; 2, a well-developed dorsal ostial microtrichial patch with short spicules; 3, a ventral ostial microtichial patch; 4, a broad ventral field of fine microspicules that extends to the flagellar plate; and 5, a relatively large flagellar plate. Thus it would appear that *Mecyclothorax punctipennis* also represents the generalized mainland species that is most similar to any extant Tahitian taxon, and therefore the most likely candidate to have colonized the Society Islands.

If *Mecyclothorax punctipennis* spawned the colonizing propagules that founded radiations on both Hawaiian and Society archipelagoes, were these radiations founded in a stepping–stone like manner from a source in the southwest Pacific? Such a stepping-stone pattern of colonization has been shown for the dominant Hawaiian Ohi`a, *Metrosideros polymorpha* (Myrtaceae) and allied species, with dispersal stemming from New Zealand and Lord Howe Island to the Marquesas and the Societies, and ultimately to the Hawaiian Islands (Wright et al. 2001; Percy et al. 2008). Elytral configuration in the two *Mecyclothorax* radiations argues against such a conclusion. In the Tahitian *Mecyclothorax striatopunctatus* species group, as well as most other Society Island *Mecyclothorax*, the eighth elytral interval just laterad the seventh stria is developed into a subcarinate margin that extends from the elytal apex to well anterad the position of the subapical elytral seta (Fig. 7E). This subcarinate ridge may be developed further in Society species, taking the form of a carinate ridge that extends anterad various lengths on the elytron (Figs 2A, B). This carinate condition has evolved several times during the evolution of Moriomorphini (Liebherr 2011b). Both the subcarinate and elongate carinate conditions were derived from the more briefly subcarinate eighth interval observable in *Mecyclothorax punctipennis*, where the distinct mesal margin of the eighth interval does not extend appreciably anterad the subapical setal depression (Fig. 7D). This brief, marked depression of the eighth interval occurs in spite of the generally shallow lateral elytral striae characteristic of this species (Fig. 7A). Several other Austral–Pacific *Mecyclothorax* species share the elytral configuration observed in *Mecyclothorax punctipennis*: *Mecyclothorax ambiguus* and *Mecyclothorax lophoides* (Chaudoir) of Australia; *Mecyclothorax basepunctus* Louwerens and *Mecyclothorax lissus* (Andrewes) of Java; and *Mecyclothorax rotundicollis* (White) plus *Mecyclothorax oopteroides* Liebherr and Marris (2009) from New Zealand. Therefore colonization of the Societies has been followed during diversification by evolutionary enhancement of this carina (Fig. 7E). Conversely in the Hawaiian *Mecyclothorax montivagus*, this carina is less developed than in *Mecyclothorax punctipennis*; i.e. the eighth interval is more rounded mesally, with its margin most distinct, though still broadly rounded, laterad the subapical setal depression (Fig. 7F). Thus the Hawaiian and Australian taxa share the absence of a subcarinate interval extension anterad the subapical seta. Based on the pattern of evolutionary transformation in this character, whereby the Australian condition is considered plesiomorphic (Fig. 7D), evolution has proceeded independently in two directions; 1, toward more carinate elytra in the Societies (Fig. 7E); and 2, to a less carinate, more rounded eighth interval in Hawaii (Fig. 7F). Given this pattern, it is most parsimonious to hypothesize two independent colonization events involving the same colonizing species, with subsequent independent radiations in the Tahitian and Hawaiian archipelagoes.

Independent colonization of the Societies and Hawaii by propagules derived from populations of *Mecyclothorax punctipennis* suggests several ancillary conclusions. First, both colonizing events would seem to have involved propagules derived from one of the currently most common carabid beetle species in Australia, *Mecyclothorax punctipennis*. The numerical dominance and ecological plasticity of this species, which occurs from dry *Xanthorrhea*-*Eucalyptus* forest at sea level to open montane snow gum (*Eucalyptus pauciflora* Sieber) forests at over 1500 m elevation (unpubl. data), enhances the probability that were a propagule produced from this species, the colonizers would possess substantial genetic variability to allow survival as ecological pioneers across the range of habitats occupied by the beetles in Australia. This range would include the present–day leeward montane shrubland habitat of *Mecyclothorax montivagus* in Maui.

Second, if *Mecyclothorax punctipennis* were the source of colonizing propagules, colonization of both the Society and Hawaiian Islands must have occurred recently. Present evidence for the Society radiation, with species restricted to Moorea and Tahiti, would place the age of origin of the fauna at no more than 2.25 Ma were the radiation founded on Moorea, or 1.75 Ma if founded on Tahiti (Craig et al. 2001). In Hawaii, the most generalized species, *Mecyclothorax montivagus*, occurs on Haleakala, a volcano that completed the shield–building stage by 0.95 Ma (Sherrod et al. 2003). In Hawaii, there are no *Mecyclothorax* in Kauai, and the Oahu taxa have sister groups on Maui Nui (Liebherr 2009a; Liebherr and Krushelnycky 2011). This places Maui Nui, and most defensibly Haleakala based on extant, observable taxa, as the point of original *Mecyclothorax* colonization in Hawaii. Thus both island radiations have evolved over the past 1–2 Myr. Bocher (1995) found that nearly all of the 140 carabid beetle species assembled in 2 Myr old glacial deposits at Kap København, North Greenland remain extant. If the widespread, ecologically plastic *Mecyclothorax punctipennis* responded to climatic change as did the broadly distributed Holarctic coleopteran species that tracked Pleistocene climate change (Coope 1979), then a 2 Myr species duration for *Mecyclothorax punctipennis* seems a reasonable working hypothesis.

Finally, as the Tahitian and Hawaiian radiations have the same, extant sister species as their adelphotaxon, intense acceleration of the speciation rate is demonstrable in both island archipelagoes. This accelerated diversification must be directly attributable to life on these isolated volcanic islands. The subtropical Hawaiian and Society volcanic islands developed forests quickly, as orographic rainfall synergized nutrient transfer to forest plants within thousands of years of plant colonization (Kitayama et al. 1997; Vitousek et al. 1997). Evolutionary loss of flight wings in both of these island radiations enhanced speciation rate in the flightless *Mecyclothorax* by reducing gene flow among populations (Liebherr 1988), thereby facilitating adaptation to specific locales (Darlington 1943; Southwood 1977). Allopatric speciation among these poorly connected populations was facilitated initially by range fragmentation caused by by emplacement of new lava flows on the landscape resulting in isolated forest kipukas (Zimmerman 1948). Upon cessation of major volcanic activity, rampant erosion and valley formation isolated organisms in islands or peninsulas of montane forest habitat. The number of ecological components in these habitats was restricted by the colonization process, and those taxa present faced reduced competition for resources except among phylogenetic relatives that shared their general way of life. This restrictive competition favored subsequent specialization within what remained a more generalized way of life in the mainland source community. In the Tahitian *Mecyclothorax* fauna, this has resulted in species that have specialized to live predominantly in leaf litter, such as the large-bodied *Mecyclothorax muriauxi* species group (Perrault 1984) and others that are observed nearly always in arboreal microhabitats, such as species of the small-bodied *Mecyclothorax globosus* group (Perrault 1989). This ecological divergence took place without the ability to colonize remote habitat patches via winged flight. However, forest habitats anastomosed through time as lava flows became forested, allowing beetles to colonize newly available habitats terrestrially, with subsequent lava flows dissecting the landscape in new and different ways. The beetles’ various ecological specializations combined with their aggregate role as one of the dominant predatory lineages, ensuring that these radiating insects persisted in the many historically colonized forest patches, thereby setting the stage for multiplicative generation of ever more allopatric species; an island variant of Noonan’s (1988) continental cyclic vicariance. This multiply-layered *Mecyclothorax* fauna, with many sympatric species representing different sublineages of the radiation, prompted Perrault (1992) to suggest that Tahiti was colonized repeatedly by numerous waves of different *Mecyclothorax* colonists. However, the presence of a very similar pattern of intense levels of sympatry and extreme specialization in the much more isolated Hawaiian Islands, coupled with the extreme similarity of only the mainland *Mecyclothorax punctipennis* to any of the Hawaiian *Mecyclothorax*, supports evolution from a single colonization event in Hawaii. In the Society Islands, moreover, the presence of species assignable to *Mecyclothorax* based on appropriate generic–level characters, yet exceedingly different (e.g., Fig. 2B) from any other *Mecylothorax* species present anywhere beyond the Society Islands, suggests that diversification has proceeded apace within this archipelago to produce today’s incredibly diverse fauna.

## Supplementary Material

XML Treatment for Mecyclothorax perraulti

XML Treatment for Mecyclothorax pahere

XML Treatment for Mecyclothorax menemene

XML Treatment for Mecyclothorax mahatahi

XML Treatment for Mecyclothorax popotioaoa

XML Treatment for Mecyclothorax mapo

XML Treatment for Mecyclothorax fatata

## References

[B1] Baehr M (1998) A preliminary survey of the classification of Psydrinae (Coleoptera: Carabidae). In Ball GE, Casale A, Vigna Taglianti V (Eds) Phylogeny and classification of Caraboidea (Coleoptera: Adephaga). Proceedings of a symposium (28 August, 1996, Florence, Italy). 20 International Congress of Entomology, Atti Museo Regionale di Scienze Naturali (Museo Regionale di Scienze Naturali–Torino, Torino), 359–368.

[B2] Baehr M (2004) The Amblytelini, a tribe of corticolous ground beetles from Australia: taxonomy, phylogeny, biogeography. (Coleoptera: Carabidae: Psydrinae). Coleoptera 8: 1-238.

[B3] Baehr M (2009) A new species of the genus *Mecyclothorax* Sharp from New South Wales (Insecta: Carabidae: Psydrinae). Records of the Australian Museum 61: 89-92. doi: 10.3853/j.0067-1975.61.2009.1519

[B4] Baehr M Lorenz W (1999) A reevaluation of *Loeffleria globicollis* Mandl from Borneo (Insecta, Coleoptera, Carabidae, Psydrinae). Spixiana 22: 263-267.

[B5] Ball GE Shpeley D (1983) The species of eucheiloid Pericalina: classification and evolutionary considerations (Coleoptrea: Carabidae: Lebiini). Canadian Entomologist 115: 743-806. doi: 10.4039/Ent115743-7

[B6] Blackburn T (1892) Notes on Australian Coleoptera, with descriptions of new species. Proceedings of the Linnean Society of New South Wales (Ser. 2) 6: 479–550.

[B7] Bocher J (1995) Paleoentomology of the Kap København Formation, a Plio–Pleistocene sequence in Peary Land, North Greenland. Meddelelser om Grønland, Geoscience 33: 82 pp.

[B8] Bouchard P Bousquet Y Davies AE Alonzo–Zarazaga MA Lawrence JF Lyal CHC Newton AF Reid CAM Schmitt M Ślipiński SA Smith ABT (2011) Family–group names in Coleoptera (Insecta). Zookeys 88: 1-972. doi: 10.3897/zookeys.88.807 PMC3088472 21594053

[B9] Britton EB (1948) A revision of the Hawaiian species of *Mecyclothorax* (Coleoptera: Carabidae). Occasional Papers of Bernice P. Bishop Museum 19: 107-166.

[B10] Coope GR (1979) Late Cenozoic fossil Coleoptera: evolution, biogeography, and ecology. Annual Review of Ecology and Systematics 10: 247-267. doi: 10.1146/annurev.es.10.110179.001335

[B11] Craig DA Currie DC Joy DA (2001) Geographical history of the central–western Pacific black fly subgenus *Inseliellum* (Diptera: Simuliidae: *Simulium*) based on a reconstructed phylogeny of the species, hot–spot archipelagoes and hydrological considerations. Journal of Biogeography 28: 1101-1127. doi: 10.1046/j.1365-2699.2001.00619.x

[B12] Crampton HE (1917[1916]) Studies on the variation, distribution, and evolution of the genus *Partula*, the species inhabiting Tahiti. Washington, DC, The Carnegie Institution of Washington, 313 pp. + 34 pls.

[B13] Crampton HE (1932) Studies on the variation, distribution, and evolution of the genus *Partula*, the species inhabiting Moorea. Washington, DC, Carnegie Institute of Washington, vi + 335 pp. + 8 pls.

[B14] Darlington PJ Jr (1936) Variation and atrophy of flying wings of some carabid beetles. Annals of the Entomological Society of America 24: 136-179.

[B15] Darlington PJ Jr (1943) Carabidae of mountains and islands: data on the evolution of isolated faunas, and on atrophy of wings. Ecological Monographs 13: 37-61. doi: 10.2307/1943589

[B16] Deuve T (1987) Descriptions de deux carabiques nouveaux de Nouvelle–Calédonie et de Thaïlande (Coleoptera, Caraboidea, Psydridae, Trechidae). Revue Française d’Entomologie (NS) 9: 143-146.

[B17] Faichney IDE Webster JM Clague DA Paduan JB Fullagar P (2010). Unraveling the tilting history of the submerged reefs surrounding Oahu and the Maui Nui Complex, Hawaii. Geochemistry Geophysics Geosystems 11(7): 20 pp doi: 10.1029/2010GC003044

[B18] Giambelluca TW Schroeder TA (1998) Climate. In Juvik SP, Juvik JO (Eds) Atlas of Hawaii. Honolulu, University of Hawaii Press, 49–59.

[B19] Guillou H Blais S Guille G Legendre C Maury RC Caroff M. Cotten J (2000) Unspiked K–Ar dating of the subaerial volcanic activity of Moorea, Huahine, Raiatea, Bora Bora and Maupiti (Society Islands). EOS, Transactions of the American Geophysical Union 81(48): F1369.

[B20] Hoch H (2006) New Cixiidae from eastern Polynesia: *Oteana* gen. nov. and *Manurevana* gen. nov. (Hemiptera: Fulgoromorpha). Zootaxa 1209: 1-47.

[B21] Jeannel R (1940) III. Coléoptères. Croisière du Bougainville aux îles australes Françaises. Mémoires du Muséum National d’Histoire Naturelle 14: 63-201.

[B22] Jeannel R (1943) Un carabique nouveau de la Nouvelle–Calédonie. Revue Française d’Entomologie 10: 84-86.

[B23] Kitayama K Schuur EAG Drake DR Mueller–Dombois D (1997) Fate of a wet montane forest during soil ageing in Hawaii. Journal of Ecology 85: 669-679. doi: 10.2307/2960537

[B24] Liebherr JK (1988) Gene flow in ground beetles (Coleoptera: Carabidae) of differing habitat preference and flight-wing development. Evolution 42: 129-137. doi: 10.2307/2409121 28563855

[B25] Liebherr JK (2006[“2005”]) New species of *Mecyclothorax* (Coleoptera: Carabidae,Psydrini) from Polipoli,Maui define an area of endemism on Haleakala Volcano, Hawaii. Journal of the New York Entomological Society 113: 97–128. doi: 10.1664/0028-7199(2005)113[0097:NSOMCC]2.0.CO;2

[B26] Liebherr JK (2007[“2006”]) Taxonomic revision of the *Mecyclothorax* beetles (Coleoptera: Carabidae, Psydrini) of Molokai, Hawaii and recognition of areas of endemism on Kamakou volcano. Journal of the New York Entomological Society 114: 179–281. doi: 10.1664/0028-7199(2007)114[179:TROTMB]2.0.CO;2

[B27] Liebherr JK (2008) Taxonomic revision of *Mecyclothorax* Sharp (Coleoptera, Carabidae) of Hawaii Island: abundant genitalic variation in a nascent island radiation. Deutsche Entomologische Zeitshrift 55: 19-78. doi: 10.1002/mmnd.200800004

[B28] Liebherr JK (2009a) Taxonomic revision of the *Mecyclothorax* beetles (Coleoptera: Carabidae) of Oahu: epithets as epitaphs for an endangered fauna? Systematic Entomology 34: 649–687. doi: 10.1111/j.1365-3113.2009.00477.x

[B29] Liebherr JK (2009b) Native and alien Carabidae (Coleoptera) share Lanai, an ecologically devastated island. The Coleopterists Bulletin 63: 383-411. doi: 10.1649/1176.1

[B30] Liebherr JK (2011a) The *Mecyclothorax* beetles (Coleoptera: Carabidae: Moriomorphini) of West Maui, Hawaii: taxonomy, biogeography, and conservation. Deutsche Entomologische Zeitschrift 58: 15-76. doi: 10.1002/mmnd.201100005

[B31] Liebherr JK (2011b) Cladistic assessment of subtribal affinities within the tribe Moriomorphini with description of *Rossjoycea glacialis*, gen. n. and sp. n. from the South Island, and revision of *Meonochilus* Liebherr and Marris from the North Island, New Zealand (Coleoptera, Carabidae). ZooKeys 147: 277-335. doi: 10.3897/zookeys.147.1898 PMC3286249 22371667

[B32] Liebherr JK (in press) New *Mecyclothorax* spp. (Coleoptera: Carabidae: Moriomorphini) define Mont Mauru, eastern Tahiti Nui, as a distinct area of endemism. ZooKeys. 10.3897/zookeys.227.3797 PMC3487650 23166465

[B33] Liebherr JK Krushelnycky PD (2011). *Mecyclothorax palikea* sp. n. from the Waianae Range, Oahu, and the biogeographical history of Hawaii’s *Mecyclothorax flavomarginatus* species group (Coleoptera: Carabidae: Moriomorphini). Insect Systematics & Evolution 42: 365-384. doi: 10.1163/187631211X608718

[B34] Liebherr JK Marris JWM (2009) Revision of the New Zealand species of *Mecyclothorax* Sharp (Coleoptera: Carabidae: Psydrinae, Mecyclothoracini) and the consequent removal of several species to *Meonochilus* gen. n. (Psydrinae: Meonini). New Zealand Entomologist 32: 5-22. doi: 10.1080/00779962.2009.9722172

[B35] Liebherr JK Will KW (1998) Inferring phylogenetic relationships within Carabidae (Insecta, Coleoptera) from characters of the female reproductive tract. In Ball GE, Casale A, Vigna Taglianti V (Eds) Phylogeny and classification of Caraboidea (Coleoptera: Adephaga). Proceedings of a symposium (28 August, 1996, Florence, Italy). 20 International Congress of Entomology, Atti Museo Regionale di Scienze Naturali (Museo Regionale di Scienze Naturali–Torino, Torino), 107–170.

[B36] Lindroth CH (1974) On the elytral microsculpture of carabid beetles (Col. Carabidae). Entomologica Scandinavica 5: 251-264. doi: 10.1163/187631274X00290

[B37] Maddison DR (1993) Systematics of the Holarctic beetle subgenus *Bracteon* and related *Bembidion* (Coleoptera: Carabidae). Bulletin of the Museum of Comparative Zoology 153: 143-299.

[B38] Moore BP (1984) Taxonomic notes on some Australasian *Mecyclothorax* Sharp (Coleoptera: Carabidae: Psydrinae) and descriptions of new species. Journal of the Australian Entomological Society 23: 161-166. doi: 10.1111/j.1440-6055.1984.tb01935.x

[B39] Moore BP (1985) The Carabidae of Norfolk Island. In Ball GE (Ed) Taxonomy, Phylogeny and Zoogeography of Beetles and Ants. Dr W Junk Publishers, Dordrecht, 237–256.

[B40] Moore BP (1992) The Carabidae of Lord Howe Island (Coleoptera: Carabidae). In Noonan GR, Ball GE, Stork NE (Eds) The Biogeography of Ground Beetles of Mountains and Islands. Intercept, Ltd., Andover, Hampshire, UK, 159–173.

[B41] Moore BP Weir TA Pike JE (1987) Coleoptera: Adephaga: Rhysodidae and Carabidae. Zoological Catalogue of Australia. Australian Government Printing Service, Canberra 4: 17-320.

[B42] Mueller–Dombois D Fosberg FR (1998) Vegetation of the Tropical Pacific Islands. New York, NY, Springer Verlag, xvii + 733 pp.

[B43] Noonan GR (1988) Biogeography of North American and Mexican insects, and a critique of vicariance biogeography. Systematic Zoology 37: 366-384. doi: 10.2307/2992199

[B44] Percy DM Garver AM Wagner WL James HF Cunningham CW Miller SE Fleischer RC (2008) Progressive island colonization and ancient origin of Hawaiian *Metrosideros* (Myrtaceae). Proceedings of the Royal Society B 275: 1479-1490. doi: 10.1098/rspb.2008.0191 18426752 PMC2602662

[B45] Perrault GG (1978a) La faune des Carabidae de Tahiti II. genre *Mecyclothorax* (Sharp). Nouvelle Revue d’Entomologie 8: 27-36.

[B46] Perrault GG (1978b) La faune des Carabidae de Tahiti II. Genre *Mecyclothorax* (Sharp). Nouvelle Revue d’Entomologie 8: 133-162.

[B47] Perrault GG (1984) La faune des Carabidae de Tahiti VI. révision du genre *Mecyclothorax* (Sharp) (Psydrini). 1. le groupe de *Mecyclothorax muriauxi* Perrault (Coleoptera). Nouvelle Revue d’Entomologie (NS) 1: 19-31.

[B48] Perrault GG (1986). La faune des Carabidae de Tahiti VII. Révision du genre *Mecyclothorax* (Sharp) (Psydrini). 2. les groupes de *Mecyclothorax striatopunctatus* n. sp., *Mecyclothorax dannieae* Perrault, *Mecyclothorax marginatus* Perrault et *Mecyclothorax viridis* Perrault (Coleoptera). Nouvelle Revue d’Entomologie (NS) 3: 439-455.

[B49] Perrault GG (1987) Microendemisme et speciation du genre *Mecyclothorax* (Coleoptera – Carabidae Psydrini) à Tahiti. Bulletin de la Société Zoologique de France 112: 419-427.

[B50] Perrault GG (1988) La faune des Carabidae de Tahiti. VIII. révision du genre *Mecyclothorax* Sharp (Psydrini) 3. les groups de *Mecyclothorax altiusculus* Britton et de *Mecyclothorax gourvesi* Perrault (Coleoptera). Nouvelle Revue d’Entomologie (NS) 5: 229-245.

[B51] Perrault GG (1989) La faune des Carabidae de Tahiti: IX. révision du genre *Mecyclothorax* (Sharp) (Psydrini) 4. le groupe de *Mecyclothorax globosus* Britton (Coleoptera). Nouvelle Revue d’Entomologie (NS) 6: 57-70.

[B52] Perrault GG (1992) Endemism and biogeography among Tahitian *Mecyclothorax* species (Coleoptera: Carabidae: Psydrini). In Noonan, GR, Ball GE, Stork NE (Eds), The Biogeography of Ground Beetles of Mountains and Islands. Intercept, Ltd.:, Andover, Hampshire, UK, 201–215.

[B53] Polhemus DA (2010) Eight new species of micropterous Nabidae (Heteroptera) from the Society Islands, French Polynesia, with consideration of hotspot island speciation patterns. Tijdschrift voor Entomologie 153: 53-78.

[B54] Price JP Elliott–Fisk D (2004) Topographic history of the Maui Nui Complex, Hawaii, and its implication for biogeography. Pacific Science 58: 27-45. doi: 10.1353/psc.2004.0008

[B55] Sherrod DR Nishimitsu Y Tagami T (2003) New K–Ar ages of the geological evidence against rejuvenated–stage volcanism at Haleakalā, East Maui, a post–shield–stage volcano of the Hawaiian island chain. Geological Society of America Bulletin 115: 683-694. doi: 10.1130/0016-7606(2003)115<0683:NKAATG>2.0.CO;2

[B56] Sloane TG (1890) Studies in Australian Entomology. No. IV.––new genera and species of Carabidae. Proceedings of the Linnean Society of New South Wales 5(2nd series): 641–653.

[B57] Sloane TG (1898) On Carabidae from West Australia, sent by Mr. A M Lea (with descriptions of new genera and species, synoptic tables, &c.). Proceedings of the Linnean Society of New South Wales 23: 444-520.

[B58] Southwood TRE (1977) Habitat, the templet for ecological strategies? Journal of Animal Ecology 46: 337–365. doi: 10.2307/3817

[B59] United Kingdom Hydrographic Office (2012) South Pacific Ocean, Polynésie Française, Approaches to Tahiti and Moorea, scale 1:100,000 at 17°40'S. Taunton, Somerset, UK.

[B60] Vitousek PM Chadwick OA Crews TE Fownes JH Hendricks DM Herbert D (1997) Soil and ecosystem development across the Hawaiian Islands. GSA Today 7 (9): 1-8.11541665

[B61] Wahlroos S (2002) English–Tahitian Tahitian–English Dictionary. Honolulu, Hawai`i, The Mā`ohi Heritage Press, xxvi + 684 pp.

[B62] Wright SD Yong CG Dawson JW Gardner RC (2001) Stepping stones to Hawaii: a trans–equatorial dispersal pathway for *Metrosideros* (Myrtaceae) inferred from nrDNA (ITS + ETS). Journal of Biogeography 28: 769-774. doi: 10.1046/j.1365-2699.2001.00605.x

[B63] Zimmerman EC (1948) Introduction. Insects of Hawaii 1: xliv + 206 pp (2001 reissue).

